# Ethnobotanical Heritage of Edible Plants Species in Mueang District, Yasothon Province, Northeastern Thailand

**DOI:** 10.3390/biology14091264

**Published:** 2025-09-13

**Authors:** Piyaporn Saensouk, Surapon Saensouk, Thawatphong Boonma, Yuefeng Zhang, Lingling Lv, Tammanoon Jitpromma

**Affiliations:** 1 Diversity of Family Zingiberaceae and Vascular Plant for Its Applications Research Unit, Department of Biology, Faculty of Science, Mahasarakham University, Maha Sarakham 44150, Thailand; pcornukaempferia@yahoo.com; 2 Diversity of Family Zingiberaceae and Vascular Plant for Its Applications Research Unit, Walai Rukhavej Botanical Research Institute, Mahasarakham University, Maha Sarakham 44150, Thailand; boonma.thawat@gmail.com (T.B.); zhangyuefeng_tj@163.com (Y.Z.); jitpromma.t@gmail.com (T.J.); 3 Agriculture and Food Engineering College, Baise University, Baise 533000, China; lvlingling207@163.com

**Keywords:** cultivated species, edible plants, ethnobotany, local vegetables, medicinal plants, Thailand, traditional knowledge, wild species, Yasothon Province

## Abstract

Traditional knowledge of edible plants is essential for food security, nutrition, and cultural identity in rural communities, yet many regions, including Mueang District, Yasothon Province (MY), remain underexplored in ethnobotanical studies. The lack of systematic documentation of local plant diversity and uses in MY presents a significant gap in understanding how these resources contribute to the sustainability of local food systems. This study addresses this gap by providing a comprehensive ethnobotanical survey of edible plants in Mueang District, Northeastern Thailand. Documenting 170 species across 60 families, the study reveals a rich plant diversity reflecting a dynamic interplay of native and introduced species, as well as cultivated and wild-harvested resources that sustain local food security, nutrition, and cultural traditions. Fabaceae emerged as the most species-rich family, while staple cereals such as *Oryza sativa* L. and *Zea mays* L. dominate use-value rankings. Quantitative indices, including Species Use Value (SUV), Relative Frequency of Citation (RFC), Cultural Food Significance Index (CFSI), and Fidelity Level (%FL), elucidate nuanced patterns of plant utilization, highlighting multifunctional species that bridge food and medicine. High cultural consensus on key medicinal plants underscores the deep integration of traditional healthcare within local food systems. The diversity of edible plant uses—from beverages and condiments to medicinal foods—illustrates adaptive strategies that enhance dietary diversity and ecological resilience. These findings contribute to the understanding of biocultural diversity and underscore the importance of conserving both plant biodiversity and indigenous knowledge to promote sustainable, locally adapted food systems. The study also identifies priority species for future pharmacological and agronomic research to support climate-resilient food sovereignty.

## Simple Summary

This study explores the wide species of edible plants used by people in Mueang District, Yasothon Province (MY), Northeastern Thailand, where little research has been done before. We found 170 different plant species that local communities rely on for food, medicine, and cultural traditions. Both wild and farmed plants, as well as native and introduced species, play important roles in supporting local diets and health. Some plants have multiple uses, providing both nutrition and healing benefits. By documenting this traditional knowledge, the study highlights the importance of protecting plant diversity and cultural heritage. This information can help support sustainable food systems and guide future research to improve food security and well-being in the region.

## 1. Introduction

Traditional knowledge of edible plants plays a crucial role in maintaining food security, nutrition, and cultural identity, particularly among rural communities that rely on both cultivated and wild resources [1]. For generations, people living in close connection with their natural environments have developed deep and practical knowledge of edible plants, encompassing species identification, harvesting techniques, preparation methods, and seasonal availability [2]. This knowledge, passed down orally and through practice, is not only culturally significant but also ecologically adaptive, providing diverse and nutrient-rich diets that support local resilience [3].

Ethnobotanical studies that document such knowledge are essential for biodiversity conservation and sustainable food system development, especially in the face of rapid environmental and social change [4]. Climate change, land-use transformation, and agricultural intensification are accelerating the loss of wild plant species and eroding traditional knowledge systems. This trend poses serious consequences: the disappearance of edible plant knowledge reduces dietary diversity, undermines health and nutrition, and weakens community resilience to climate and market shocks. In the long term, failure to preserve ethnobotanical heritage risks cultural homogenization, nutritional insecurity, and the irreversible loss of biocultural diversity [5].

Globally, ethnobotany has emphasized the cultural and ecological significance of local food systems. Foundational works by Alexiades [6,7] and Voeks [8,9,10] advanced quantitative and methodological approaches that linked plant use with biodiversity conservation, livelihoods, and resilience. In Southeast and South Asia, research from Bangladesh, Laos, and Myanmar [11,12,13] has highlighted the dietary and cultural importance of edible plants, showing that they are not minor supplements but integral to subsistence strategies and nutritional security. These studies confirm the role of edible plants in maintaining cultural identity across diverse ecological and social contexts.

Despite these insights, methodological and regional limitations persist. Many surveys in Asia focus on medicinal plants [14,15], while edible plants receive less systematic attention. Existing studies often report descriptive species lists and rely primarily on the Use Value (UV) index, neglecting complementary measures such as Relative Frequency of Citation (RFC), Cultural Food Significance Index (CFSI), or Family Use Value (FUV) [16]. Small sample sizes, limited geographic coverage, and reliance on qualitative observations further constrain broader applicability. In Thailand, most ethnobotanical work has focused on northern and northeastern regions [17,18], leaving other areas underexplored. Few studies integrate multiple indices, which would allow a more holistic understanding of plant use and cultural significance.

Thailand is also a major producer of staple crops. In 2022, the country produced 34.3 million metric tonnes of rice (a 4.06% increase from the previous year) and 4.9 million metric tonnes of maize (a 0.99% increase from 2021) [19,20]. While such figures highlight the significance of agricultural production for rural communities, they also contrast with the continued reliance on wild and traditional edible plants in MY, where local knowledge remains central to food security, nutrition, and cultural identity.

Given this context, our study addresses the following research questions: (1) What is the diversity and composition of edible plants currently used in MY, and how are they sourced (cultivated or wild)? (2) How do local people categorize and assign meaning to different edible plants across functional use groups (e.g., vegetables, fruits, beverages, and condiments)? (3) Which species are culturally most significant, and how can ethnobotanical indices help quantify that importance?

To answer these questions, this study aims to document the traditional knowledge and ethnobotanical uses of edible plants in MY. This includes (1) identifying the species used as food, whether wild or cultivated; (2) recording the parts of plants used and preparation methods; (3) categorizing plant uses across various functional groups (e.g., vegetables, fruits, spices, beverages, and foods); and (4) analyzing their relative cultural importance using standard ethnobotanical indices such as Species Use Value (SUV), Relative Frequency of Citation (RFC), Cultural Food Significance Index (CFSI), and Fidelity Level (FL).

These indices quantify distinct aspects of traditional plant knowledge. SUV reflects species versatility by the average number of uses [21], RFC measures prominence based on informant mentions [22], CFSI provides a multidimensional assessment of frequency, availability, and cultural context [23], and FL compares family-level contributions to local food knowledge [24]. By integrating these measures, the study offers a multifaceted approach to assess edible plant diversity and cultural relevance in MY, contributing to ethnobotanical research in Thailand and supporting preservation of traditional ecological knowledge amid rapid socio-environmental change.

## 2. Materials and Methods

### 2.1. Study Area

**Figure 1:**
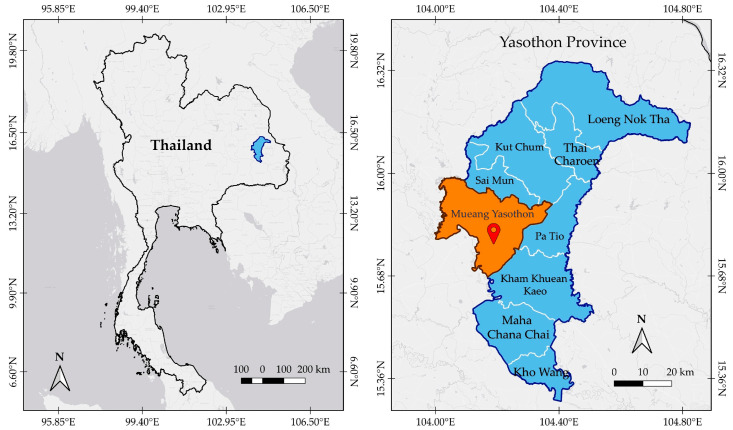
Map of the study area: Left shows Yasothon Province in blue within Thailand. The right highlights MY in orange within the province. (map created with “QGIS” program ver. 3.34 [26], geographic system ID: WGS 84, EPSG 4326 designed by Phiphat Sonthongphithak).

The study was conducted in MY, the administrative and economic center of Yasothon Province, located in the southern part of the Khorat Plateau in Northeastern Thailand (Isan region), a region characterized by a semi-arid climate, distinct wet and dry seasons, low-lying terrain, and a mosaic of paddy fields, upland areas, village groves, seasonal wetlands, as well as mixed deciduous and dry dipterocarp forests (Figure 1) [25].

Mueang District spans a variety of landscapes, including rice paddies, home gardens, secondary forests, and upland fields, which support a wide diversity of native and cultivated plant species. The district is home to ethnic Lao Isan communities, whose traditional knowledge of edible plants has been shaped over generations through close interaction with their environment.

With a population that relies heavily on subsistence agriculture, foraging, and small-scale farming, local communities in Mueang District maintain a rich ethnobotanical heritage. Seasonal changes, cultural practices, and Buddhist festivals also influence plant use and harvesting patterns. This district serves as a representative area for studying traditional ecological knowledge in the broader Isan region, where cultural identity and biodiversity are tightly interwoven.

Fieldwork was focused on selected villages and surrounding rural areas within the district, where elders, farmers, foragers, and local healers provided insights into the use, management, and conservation of edible plant species. These locations were chosen based on their accessibility, diversity of habitats, and the willingness of communities to participate in the documentation of their ethnobotanical knowledge.

### 2.2. Data Collection

Data collection took place over a 12-month period, from June 2024 to May 2025, to capture comprehensive insights into the diversity and ethnobotanical applications of edible plants in MY. A mixed-methods approach was adopted, combining both qualitative and quantitative research techniques.

For the purpose of this study, “edible plants” are defined as species used by the local communities for direct human consumption, including parts such as fruits, leaves, seeds, roots, and stems, either in raw, cooked, or processed forms. These plants were selected based on their culinary and medicinal uses, as reported by the informants.

Field surveys were conducted across diverse settings, including natural forests, home gardens, local markets, and communal areas—environments where edible plants are commonly encountered, cultivated, or traded. These locations were intentionally chosen to represent the ecological and cultural diversity of the region. During the fieldwork, 50 informants—comprising traditional healers, household members, and plant vendors—participated in semi-structured interviews aimed at documenting traditional knowledge and cultural significance related to Annonaceae use.

Simultaneously, botanical identification was performed through specimen collection and direct field observation. Scientific names were authenticated with assistance from local botanists and cross-referenced using the authoritative online resources, including Plants of the World Online (POWO) [27], the QBG Herbarium, and the Muséum national d’Histoire Naturelle. All confirmed specimens were deposited at the Vascular Plant Herbarium, Mahasarakham University (VMSU) in Kantharawichai District, Maha Sarakham Province, Thailand, for archival and future study.

### 2.3. Utilization Study

Ethnobotanical information on edible plant species in MY was obtained through semi-structured interviews with 100 local informants—comprising an equal number of males and females—from various districts. Informants were selected using purposive and snowball sampling techniques, aiming to include individuals with diverse backgrounds in terms of age, livelihood, and levels of traditional plant knowledge. All participants were long-term residents and homeowners in the province; those living in temporary or rented housing were excluded to ensure familiarity with local traditions. The age of participants ranged from 20 to 70 years, including elders recognized for their cultural knowledge, as well as younger adults.

Prior to the interviews, the study’s purpose was clearly communicated, and prior informed consent was obtained in accordance with the International Society of Ethnobiology (ISE) Code of Ethics [28] and principles of the Nagoya Protocol on Access and Benefit Sharing [29]. Participants were fully informed of their rights, including voluntary participation and the option to withdraw at any stage. The interviews focused on capturing traditional knowledge, such as local plant names, utilized plant parts, preparation techniques, and their respective uses.

As the research did not involve collecting sensitive personal information, formal institutional ethical approval was not required. Nevertheless, the study strictly adhered to international ethical standards for ethnobotanical research (ISE Code of Ethics; Nagoya Protocol compliance recommendations) to ensure respect, transparency, and reciprocity toward participating communities.

### 2.4. Data Analysis

To quantify ethnobotanical knowledge and plant use in MY, we applied several established indices that capture the importance, frequency, and cultural significance of edible plants. All indices were calculated based on the number of informants citing each species, the diversity of plant parts used, and the frequency of use across categories.

#### 2.4.1. Species Use Value (SUV)

The Species Use Value (SUV) is a quantitative measure used to determine how important each edible plant species is to the local community in MY. It considers both how frequently a species is used and the variety of its applications.

Following Hoffman and Gallaher [21], SUV is calculated as

(1) $$\mathrm{SUV}=\frac{\sum {\mathrm{UV}}_{\mathrm{is}}}{n_{i}}$$

where UV_is_ is the number of use reports for a particular species by an individual informant and n_i_ is the total number of informants who mentioned that species.

This formula averages the number of use reports across all informants, reflecting both the extent of use (how many people use it) and the diversity of uses (how many different ways it is used). A higher SUV indicates that the species is more widely recognized and valued within the community.

#### 2.4.2. Family Use Value (FUV)

The Family Use Value (FUV) measures the overall importance of a plant family in the local community of MY. It provides a standardized way to assess how culturally significant a family is by taking into account the use values of all species within that family.

Following Hoffman and Gallaher [21], FUV is calculated as

(2) $$\mathrm{FUV}=\frac{\sum {\mathrm{UV}}_{s}}{n_{s}}$$

where UV_s_ is the the Species Use Value (SUV) of each species in the family and n_i_ is the total number of species in that family.

By averaging the use values of all species in a family, FUV shows which plant families are most widely used and valued by the local community. A higher FUV indicates greater overall cultural importance and helps identify families that play a key role in traditional knowledge, culinary practices, and daily market use.

#### 2.4.3. Relative Frequency of Citation (RFC)

The Relative Frequency of Citation (RFC) indicates how commonly a particular plant species is mentioned by informants in the survey. It provides a simple measure of the species’ recognition and use within the community.

RFC is calculated as [22]

RFC = FC/N(3)

where FC is the number of informants who mentioned the species, N is the total number of informants interviewed, and RFC values range from 0 to 1. A higher RFC means that the species is more widely known or frequently used in the local community.

#### 2.4.4. Plant Part Value (PPV)

The Plant Part Value (PPV) measures how frequently different parts of plants are used in traditional practices. It shows which plant parts—such as leaves, fruits, roots, stems, or flowers—are most commonly utilized by the local community.

Following Gomez-Beloz [30], PPV is calculated as

(4) $$\mathrm{PPV}=\frac{\sum {\mathrm{RU}}_{\left(\mathrm{plant}\;\mathrm{parts}\right)}}{\sum \mathrm{RU}}\;\times \;100$$

where RU_plant parts_ is the number of use reports for a specific plant part and ∑RU is the total number of use reports for all plant parts.

PPV is expressed as a percentage. A higher PPV indicates that a plant part is more frequently used, highlighting its importance in traditional applications.

#### 2.4.5. Cultural Food Significance Index (CFSI)

In this study, the Cultural Food Significance Index (CFSI) was applied to assess the cultural relevance of Bignoniaceae species utilized for both culinary and medicinal purposes. Originally introduced by Pieroni in 2001 [23], the CFSI combines seven key indicators of food significance into a single, integrative metric, calculated as follows:

CFSI = QI × AI × FUI × PUI × MFFI × TSAI × FMRI × 10^−2^ (5)

The Quotation Index (QI) expresses the proportion of informants who cited a given species. The Availability Index (AI) reflects abundance, ranging from very common (4.0) to rare (1.0), and also considers the localization of use. The Frequency of Utilization Index (FUI) measures how often a species is consumed, ranging from more than once per week (5.0) to no longer used in the past 30 years (0.5). The Parts Used Index (PUI) evaluates the diversity of plant parts consumed, with different values assigned to roots, stems, leaves, flowers, fruits, and seeds. The Multifunctional Food Use Index (MFFI) accounts for culinary versatility, including preparation methods such as raw consumption, boiling, frying, roasting, or condiment use. The Taste Score Appreciation Index (TSAI) captures the perceived palatability of each species on a scale from 4.0 (terrible) to 10.0 (best). Finally, the Food-Medicinal Role Index (FMRI) assesses the degree to which a plant is recognized as both food and medicine, ranging from “not recognized” (1.0) to “very high” (5.0).

A detailed classification system and the corresponding index values for each component are provided in Table S4.

#### 2.4.6. Informant Consensus Factor (F_ic_)

The Informant Consensus Factor (F_ic_) measures how consistently informants agree on the use of medicinal plants within a particular category. It helps identify health conditions for which the community has a strong shared knowledge of plant remedies.

F_ic_ is calculated as [31]:

(6) $$F_{ic}=\frac{n_{ur}-\;n_{t}}{n_{ur}-1}$$

where n_ur_ is the total number of use reports for all species in a medicinal category, n_t_ is the number of species cited in that category, and F_ic_ values range from 0 to 1. A higher Fic indicates greater agreement among informants, reflecting a more focused and widely shared understanding of which plants are used for specific health concerns.

#### 2.4.7. Fidelity Level (%FL)

The Fidelity Level (FL) measures how strongly a plant species is associated with a specific medicinal use among informants. It indicates the proportion of people who use a species for the same health purpose, reflecting the specificity of its traditional application.

FL is calculated as [24]:

(7) $$\mathrm{FL}\;=\;\frac{I_{p}}{I_{u}}\;\times \;100$$

where I_p_ is the number of informants who cited the species for a particular ailment and I_u_ is the total number of informants who mentioned the species for any medicinal use.

A higher FL indicates that most informants associate the species with the same therapeutic use, showing a strong and consistent cultural knowledge of its medicinal value.

#### 2.4.8. Jaccard’s Similarity Index (JI)

To evaluate the similarity in species composition across different districts, the Jaccard Similarity Index (JI) was utilized. This index is a well-established metric for quantifying the resemblance between two datasets. It is calculated using the formula [32]:

JI = a/(a + b + c)(8)

where a is the number of species shared between both districts, b is the number of species found only in the first district, and c is the number of species found only in the second district.

To further investigate species diversity patterns, the Unweighted Pair Group Method with Arithmetic Mean (UPGMA) [33] was applied. The Jaccard Index served as the basis for generating a similarity matrix, which was then analyzed using the UPGMA clustering method and visualized as a heatmap through Past4 software (version 4.15) [34].

## 3. Results

### 3.1. Diversity of Edible Plant Species in MY

A total of 170 edible plant species belonging to 60 botanical families were recorded in MY (Table S1). The family Fabaceae contributed the highest number of species (19), followed by Cucurbitaceae (12), Zingiberaceae (10), Poaceae (8), and Apiaceae (7). Several other families also showed notable species richness, including Solanaceae and Brassicaceae (6 species each) and Apocynaceae and Phyllanthaceae (5 species each). In contrast, 27 families were represented by only a single species each, reflecting specialized or limited use within local communities. This pattern highlights both the breadth and concentration of ethnobotanical knowledge across taxonomic groups.

Of these, 90 species (52.94%) are native plants, while 79 species (46.47%) are introduced. One species (0.59%) has an uncertain origin. The distribution of these groups reflects both the biodiversity and cultural diversity of the area.

The majority of species are cultivated, accounting for 123 species (72.35%). Additionally, 17 species (10.00%) are both cultivated and found in the wild, while wild plants alone represent 30 species (17.65%). This distribution highlights the important role of cultivation alongside foraging in local food systems.

The detailed distribution of species richness across all recorded families is presented in Table S1.

### 3.2. Species Use Value (SUV) of Edible Plant Species in MY

The Species Use Value (SUV), a quantitative index reflecting the frequency and intensity of use reported by informants, ranged from 0.18 to 1.00 (Table S1). The highest SUV of 1.00 was recorded for *Oryza sativa*, underscoring its cultural and dietary centrality as the primary staple crop. This was followed by *Zea mays* (0.96), *Citrus × aurantiifolia* (0.94), *Allium sativum* (0.92), and *Saccharum officinarum* (0.92), all of which are key components in the local culinary system and often cultivated in household gardens.

A total of 26 species (15.29%) exhibited high SUV values (≥0.80), indicating widespread and consistent use across the community. These high-use species span multiple use categories, including beverages (e.g., *Saccharum officinarum*, 0.92), traditional condiments (e.g., *Coriandrum sativum*, 0.90), fermented or preserved foods (e.g., *Allium cepa*, 0.90), fruits (e.g., *Mangifera indica*, 0.90), staple foods (*Oryza sativa*, 1.00), sweets, desserts, or snacks (e.g., *Zea mays*, 0.96), and cultivated vegetables (e.g., *Capsicum annuum*, 0.92).

The remaining 94 species (55.29%) fell into the medium-use category (SUV 0.50–0.79). Representative examples include beverages (e.g., *Cocos nucifera*, 0.78), condiments and flavorings (e.g., *Cymbopogon citratus*, 0.74), fermented or preserved foods (e.g., *Allium tuberosum*, 0.76), fruits (e.g., *Psidium guajava*, 0.70), medicinal edible plants (e.g., *Curcuma longa*, 0.64), sweets, desserts, or snacks (e.g., *Ipomoea batatas*, 0.74), and vegetables (e.g., *Amaranthus viridis*, 0.66).

Conversely, species with low SUV values (<0.50) accounted for 50 species (29.42%) of the total recorded taxa. These include beverages (e.g., *Passiflora edulis*, 0.36), condiments and flavorings (e.g., *Zingiber officinale*, 0.38), fermented or preserved foods (e.g., *Vietnamosasa ciliata*, 0.48), fruits (e.g., *Ziziphus mauritiana*, 0.42), medicinal edible plants (e.g., *Andrographis paniculata*, 0.32), sweets, desserts, or snacks (e.g., *Vigna radiata*, 0.48), and vegetables (e.g., *Colocasia esculenta*, 0.44).

### 3.3. Family Use Value (FUV) of Edible Plant Species in MY

A total of 60 plant families were recorded for their edible uses in MY (Table 1). The Family Use Value (FUV), which represents the average use value of species within each family, ranged from 0.200 to 0.900, reflecting varying degrees of cultural and utilitarian importance as perceived by local informants.

Caricaceae exhibited the highest FUV (0.900), albeit represented by a single species, indicating its exceptional importance in local diets and practices. Similarly, Amaryllidaceae (0.860) and Anacardiaceae (0.840), with three and two species, respectively, also showed high average use values, suggesting the consistent and valuable contributions of their members to local food systems.

Several other families demonstrated high FUV values, including Alismataceae and Convolvulaceae (0.800 each), Hypericaceae (0.780), Cucurbitaceae (0.767), Solanaceae (0.747), and Apiaceae (0.734). These families are commonly associated with vegetables, herbs, and fruits, which are frequently consumed and widely used in traditional dishes and remedies.

Among the most species-rich families, Cucurbitaceae (12 species; FUV = 0.767) and Fabaceae (19 species; FUV = 0.601) stood out for their dual significance in both diversity and cultural relevance. Although Fabaceae had the highest species count, its moderate FUV suggests variable levels of use intensity among its species.

In contrast, families such as Connaraceae (FUV = 0.200), Vitaceae (0.220), Euphorbiaceae (0.260), and Colchicaceae (0.280) were found to have lower FUVs, indicating relatively limited local knowledge or use of these taxa.

### 3.4. Relative Frequency of Citation (RFC) of Edible Plant Species in MY

**Figure 2:**
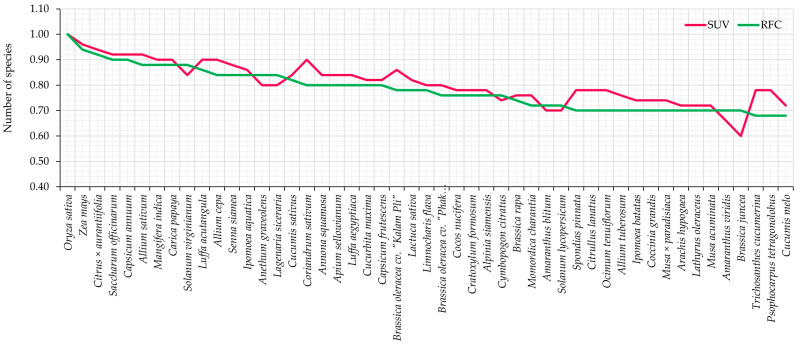
Comparison of SUV and RFC for the top 50 edible plant species recorded in MY. The figure illustrates species that are both widely known and intensively used, alongside those with specialized but high-value applications.

Relative Frequency of Citation (RFC) values range from 1.00 to 0.18, and Species Use Value (SUV) ranges from 1.00 to 0.20 (Table S1 and Figure 2). The species with the highest RFC was *Oryza sativa* (1.00), highlighting its universal recognition and vital role as a staple food in the local diet. Other frequently cited species, such as *Zea mays* (0.94), *Citrus × aurantiifolia* (0.92), *Capsicum annuum* (0.90), and *Allium sativum* (0.88), reflect their daily culinary or medicinal relevance.

At the lower end of the RFC spectrum, species such as *Gloriosa simplex* (0.28), *Phyllanthus emblica* (0.26), and *Ampelocissus martini* (0.26) were less frequently cited, suggesting either specialized knowledge or limited use among the broader community.

When comparing RFC and SUV across the top 50 species, a generally consistent pattern was observed: species that were frequently mentioned by informants often also exhibited high use values. For example, *Zea mays* (RFC = 0.94; SUV = 0.96) and *Citrus × aurantiifolia* (RFC = 0.92; SUV = 0.94) demonstrated both widespread recognition and diverse applications in food, beverages, and medicine.

However, some species exhibited notable discrepancies between RFC and SUV. *Allium sativum* (RFC = 0.88; SUV = 0.92) and *Coriandrum sativum* (RFC = 0.80; SUV = 0.90) were more intensively used than frequently mentioned, indicating their high functional value in cooking and traditional healing. In contrast, species like *Anethum graveolens* (RFC = 0.84; SUV = 0.80) and *Brassica oleracea* cv. “Kalam Pli” (RFC = 0.78; SUV = 0.86) had higher citation rates than use values, possibly due to widespread familiarity but more specific or limited practical use.

### 3.5. Utilization of Edible Plants in MY

#### 3.5.1. Edible Plants Used as Beverages

**Figure 3:**
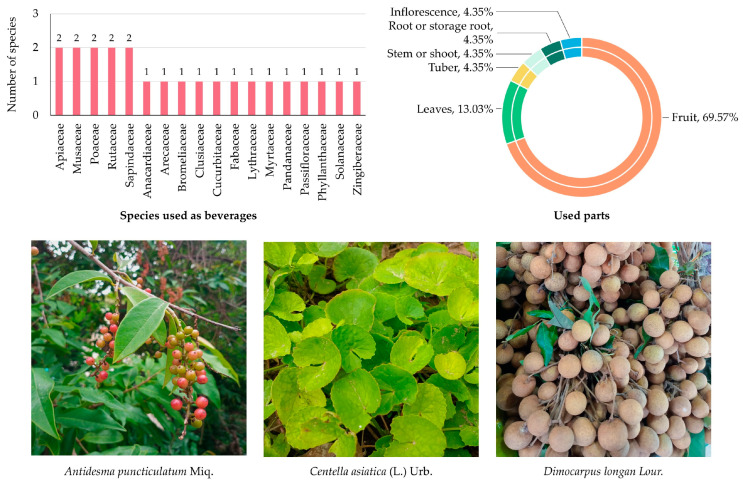
Diversity of plant species used for beverage preparation in MY. The bar chart shows the number of species by family; the circular graph shows the proportion of plant parts used. Representative species are shown below (photos by Tammanoon Jitpromma).

A total of 23 species belonging to 18 families were documented as being used in the preparation of beverages by local communities in MY (Table S1 and Figure 3). Among these, five families—Apiaceae, Musaceae, Poaceae, Rutaceae, and Sapindaceae—were each represented by two species (8.69%), highlighting their relatively greater contribution to local beverage traditions.

The remaining 13 families—Anacardiaceae, Arecaceae, Bromeliaceae, Clusiaceae, Cucurbitaceae, Fabaceae, Lythraceae, Myrtaceae, Pandanaceae, Passifloraceae, Phyllanthaceae, Solanaceae, and Zingiberaceae—were represented by one species each (4.35%), indicating a more limited yet significant role in local drink preparations.

Analysis of plant parts used in beverage preparation revealed that fruits were the most frequently utilized (16 citations; 69.57%), typically consumed as fresh juices, fermented drinks, or decoctions, underscoring their central role in traditional beverage practices. Leaves were the second most frequently used part (3 citations; 13.03%), primarily prepared as herbal teas or flavoring agents. Other parts, including inflorescences, roots or storage roots, stems, and tubers, were each reported only once (1 citation; 4.35%), reflecting occasional or species-specific applications.

#### 3.5.2. Edible Plants Used as Condiments and Flavoring

**Figure 4:**
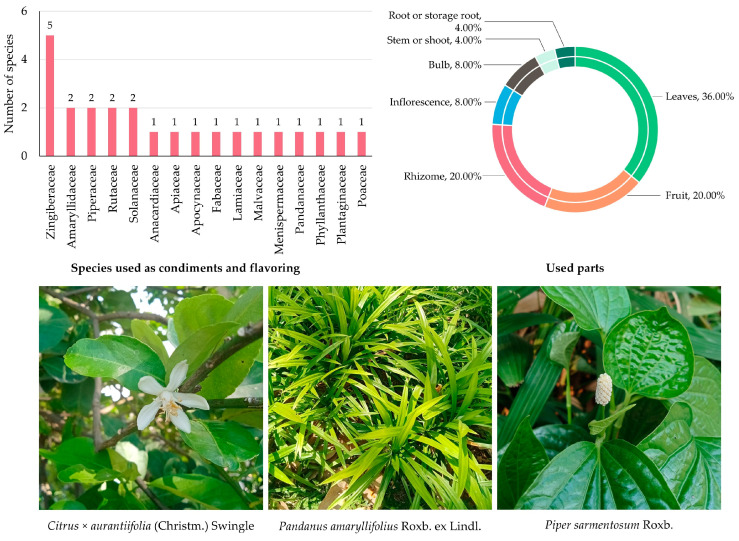
Diversity of plant species used as condiments and flavoring in MY. The bar chart shows the number of species by family; the circular graph shows the proportion of plant parts used. Representative species are shown below (photos by Tammanoon Jitpromma).

A total of 24 species belonging to 16 families were documented as being used as spices in MY (Table S2 and Figure 4). The Zingiberaceae was the most represented family, with five species (20.81%), highlighting the widespread use of gingers and related rhizomatous herbs as flavor enhancers. Families such as Amaryllidaceae, Piperaceae, Rutaceae, and Solanaceae each had two species represented (8.33%), reflecting their importance in both fresh and cooked seasoning applications. Meanwhile, other families, including Anacardiaceae, Apiaceae, Apocynaceae, Fabaceae, Lamiaceae, Malvaceae, Menispermaceae, Pandanaceae, Phyllanthaceae, Plantaginaceae, and Poaceae, were each represented by a single species (4.17%), underscoring a broad but specific spectrum of flavoring agents.

Analysis of plant parts used showed that leaves were the most frequently cited (9 citations; 36.00%), reflecting the widespread use of aromatic foliage to enhance taste and fragrance. Fruits and rhizomes were the next most important (5 citations each; 20.00%), underscoring the central role of chilies, citrus fruits, and gingers in seasoning practices. Less frequently reported parts included bulbs and inflorescences (2 citations each; 8.00%), as well as roots and stems (1 citation each; 4.00%), which were incorporated mainly for their pungent, bitter, or aromatic properties in specific preparations.

#### 3.5.3. Edible Plants Used as Fermented or Preserved

**Figure 5:**
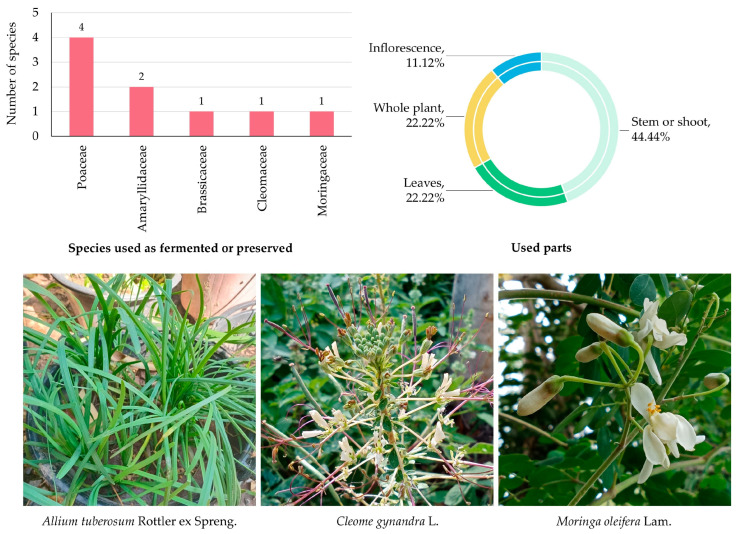
Diversity of plant species used as fermented or preserved in MY. The bar chart shows the number of species by family; the circular graph shows the proportion of plant parts used. Representative species are shown below (photos by Tammanoon Jitpromma).

A total of nine species belonging to five botanical families were identified as being used in fermented or preserved forms in MY (Table S1 and Figure 5). These preservation techniques reflect long-standing traditional knowledge that enhances food storage, flavor, and nutritional value, particularly important in rural communities with seasonal resource availability.

The Poaceae family contributed the highest number of species (4 species; 44.45%), underscoring the cultural and dietary significance of grasses and cereals—particularly rice and its derivatives—in fermented products such as rice paste, rice wine, and sour rice mixtures. Amaryllidaceae followed with two species (22.22%), while Brassicaceae, Cleomaceae, and Moringaceae each contributed one species (11.11%), mainly used in pickled or fermented vegetable preparations.

In terms of plant parts used (Figure 5), the stem was the most frequently cited (4 citations; 44.44%), reflecting the prominent role of culms and stalks in rice-based fermentation. Leaves and whole plants were each cited twice (22.22%), typically associated with leafy vegetables and herbs preserved through pickling. Inflorescences were the least reported (1 citation; 11.12%), used in more specialized preparations.

#### 3.5.4. Edible Plants Consumed as Fruits

**Figure 6:**
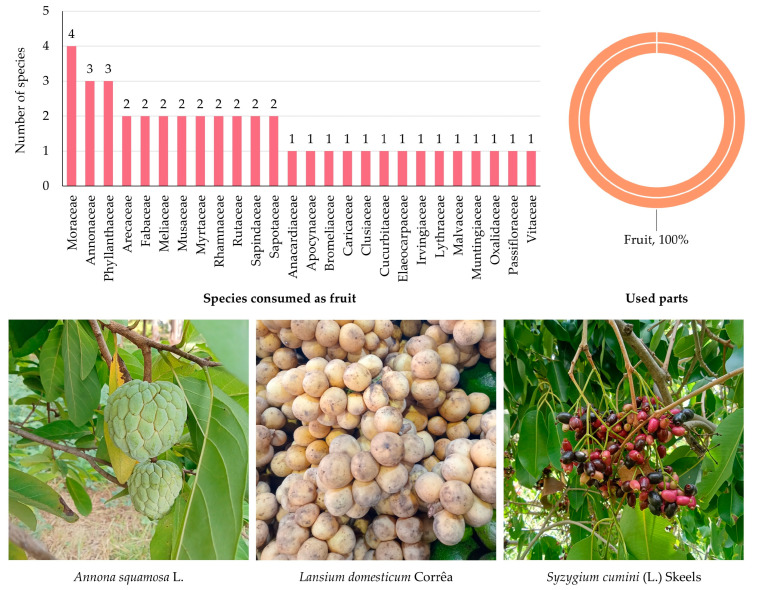
Diversity of plant species used as fruits in MY. The bar chart shows the number of species by family; the circular graph shows the proportion of plant parts used. Representative species are shown below (photos by Tammanoon Jitpromma).

A total of 42 species of edible plants used primarily for their fruits were documented, belonging to 26 botanical families (Table S1 and Figure 6). This highlights the rich diversity of fruit-bearing plants utilized in MY, reflecting both the ecological richness of the area and the cultural preference for a wide variety of seasonal and perennial fruits.

The Moraceae family contributed the highest number of fruit species (4 species; 9.54%), including culturally important and widely consumed fruits such as figs and mulberries. This was followed by Annonaceae and Phyllanthaceae, each represented by three species (7.15%). Several families, including Arecaceae, Fabaceae, Meliaceae, Musaceae, Myrtaceae, Rhamnaceae, Rutaceae, Sapindaceae, and Sapotaceae, contributed two species each (4.76%). The remaining 14 families were represented by a single species each (2.38%), underscoring the broad taxonomic spectrum of fruits incorporated into local diets.

In terms of plant parts (Figure 6), fruits were the sole part reported (42 citations; 100.00%), confirming their central role in dietary use. These fruits are consumed in diverse forms, including fresh, cooked, preserved, or processed into juices, desserts, and traditional snacks, highlighting their importance in both nutrition and cultural food practices.

#### 3.5.5. Edible Plants Used as Medicinal Edible Plants

**Figure 7:**
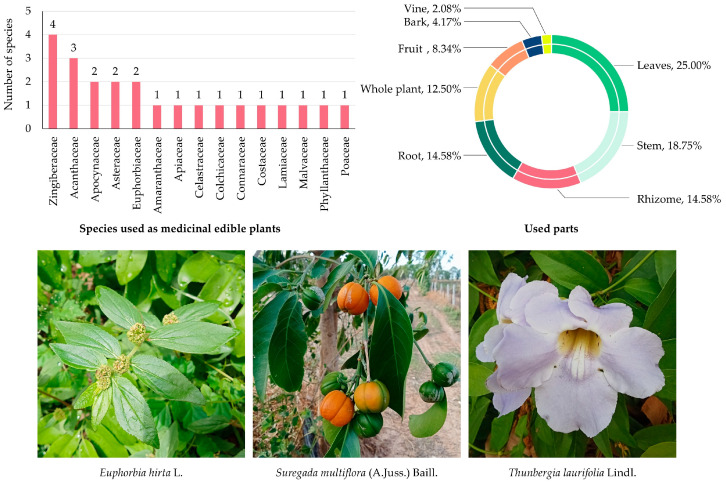
Diversity of plant species used as medicinal edible plants in MY. The bar chart shows the number of species by family; the circular graph shows the proportion of plant parts used. Representative species are shown below (photos by Tammanoon Jitpromma).

A total of 23 species from 15 botanical families were recorded as medicinal edible plants in MY (Table S1 and Figure 7). These species hold dual roles in the local community, serving both as food and as therapeutic agents, reflecting the deep-rooted integration of nutrition and health in traditional knowledge systems.

The Zingiberaceae family contributed the highest number of species (4 species; 17.39%), underscoring the central role of gingers and rhizomatous herbs in both culinary and medicinal contexts. Acanthaceae followed with three species (13.04%), while Apocynaceae, Asteraceae, and Euphorbiaceae were each represented by two species (8.69%), reflecting their recognized pharmacological properties and broad therapeutic applications. The remaining families—Amaranthaceae, Apiaceae, Celastraceae, Colchicaceae, Connaraceae, Costaceae, Lamiaceae, Malvaceae, Phyllanthaceae, and Poaceae—were each represented by one species (4.35%), highlighting a diverse but less dominant contribution.

Analysis of plant parts used (Figure 7) revealed that leaves were the most frequently reported (12 citations; 25.0%), reflecting their accessibility and wide therapeutic applicability in herbal preparations. Stems ranked second (9 citations; 18.75%), while rhizomes and roots each accounted for seven citations (14.58%), commonly employed in decoctions and infusions for treating internal ailments. Whole plants were cited in six cases (12.50%), typically when multiple organs were believed to provide synergistic effects. Fruits contributed four citations (8.34%), often consumed directly for gastrointestinal and respiratory benefits. Less frequently used plant parts included bark (2 citations; 4.17%) and vine (1 citation; 2.08%), reflecting more specialized or limited medicinal applications.

#### 3.5.6. Edible Plants Used as Staple Food

**Figure 8:**
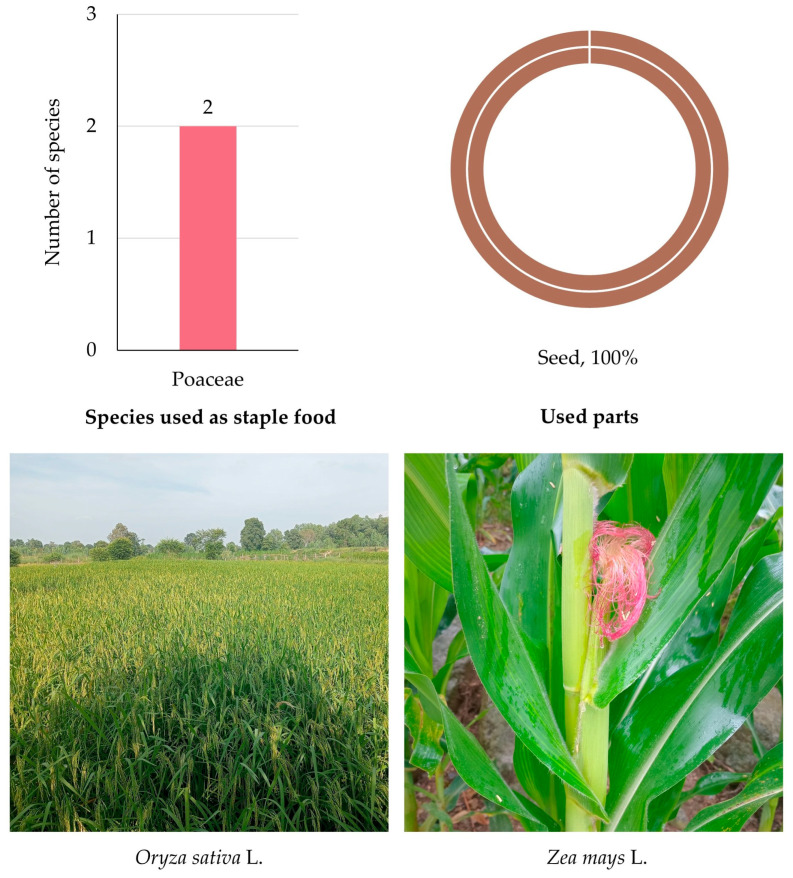
Diversity of plant species used as staple food in MY. The bar chart shows the number of species by family; the circular graph shows the proportion of plant parts used. Representative species are shown below (photos by Tammanoon Jitpromma).

Two important cereal species from the Poaceae family (100%)—*Oryza sativa* (rice) and *Zea mays* (maize)—were identified as edible plants in MY (Table S1 and Figure 8). Both species are central to local diets, serving not only as staple foods but also as ingredients in snacks, desserts, and fermented products.

The sole used plant part reported was the seed, with 100% of citations attributed to it (Figure 8). These seeds are typically consumed after processing (e.g., boiling, steaming, fermenting, roasting, or grinding), reflecting their versatility and nutritional value.

Rice, in particular, forms the foundation of daily meals, while maize is consumed in various traditional forms such as grilled, boiled, or ground into flour.

#### 3.5.7. Edible Plants Used as Sweets, Desserts, or Snacks

**Figure 9:**
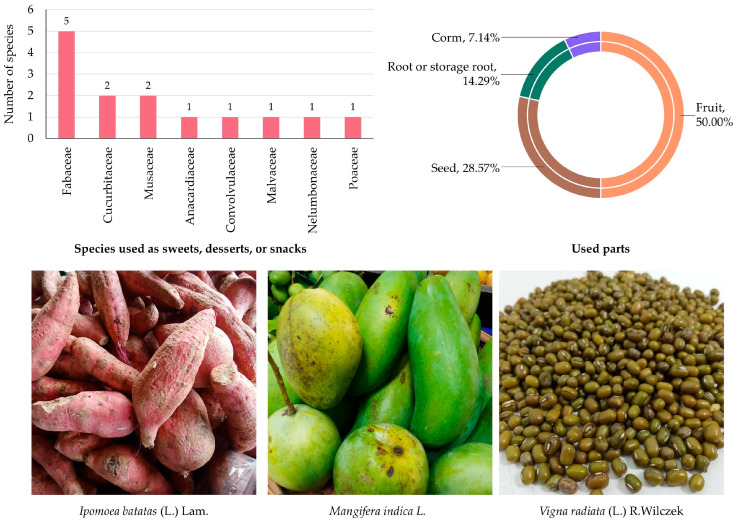
Diversity of plant species used as sweets, desserts, or snacks in MY. The bar chart shows the number of species by family; the circular graph shows the proportion of plant parts used. Representative species are shown below (photos by Tammanoon Jitpromma).

A total of 14 species from 8 botanical families were identified as being used in sweets, desserts, or snacks within MY (Table S1 and Figure 9). These plants contribute to the local culinary diversity by providing natural sweetness, texture, and flavor, often consumed as standalone treats or incorporated into traditional delicacies.

The Fabaceae family contributed the highest number of species (5 species; 35.72%), underscoring the importance of legumes and pulses as key ingredients in traditional sweets and snacks. Cucurbitaceae and Musaceae each accounted for two species (14.29%), while Anacardiaceae, Convolvulaceae, Malvaceae, Nelumbonaceae, and Poaceae were represented by one species each (7.14%).

In terms of plant parts used (Figure 9), fruits were most dominant (50.00%), reflecting their natural sweetness and versatility in desserts. Seeds contributed 28.57%, often processed into snacks, pastes, or sweetened preparations. Roots or storage roots accounted for 14.29%, while corms were the least cited (7.14%), indicating their more specialized use in local confections.

#### 3.5.8. Edible Plants Used as Vegetables

**Figure 10:**
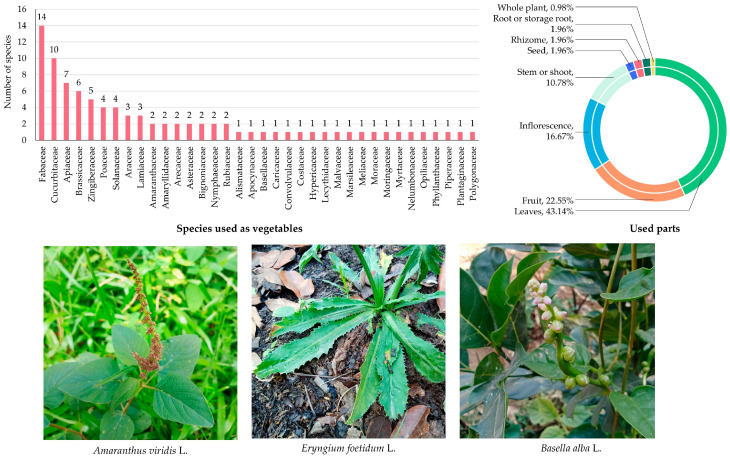
Diversity of plant species used as vegetables in MY. The bar chart shows the number of species by family; the circular graph shows the proportion of plant parts used. Representative species are shown below (photos by Tammanoon Jitpromma).

A total of 90 species belonging to 36 botanical families were documented as edible vegetables in MY (Table S1 and Figure 10), illustrating the rich diversity of plant resources incorporated into local diets. The families Fabaceae (14 species; 15.57%), Cucurbitaceae (10 species; 11.11%), Apiaceae (7 species; 7.79%), Brassicaceae (6 species; 6.68%), and Zingiberaceae (5 species; 5.57%) were the most represented, reflecting their significant roles in providing leafy greens, shoots, fruits, and rhizomes for consumption. Other families, such as Poaceae and Solanaceae, each had four species (4.44%), while Araceae and Lamiaceae contributed three species each (3.33%).

Families with two species included Amaranthaceae, Amaryllidaceae, Arecaceae, Asteraceae, Bignoniaceae, Nymphaeaceae, and Rubiaceae (each 2.22%). The remaining 20 families were represented by one species each (1.11%), including Alismataceae, Apocynaceae, Basellaceae, Caricaceae, Convolvulaceae, Costaceae, Hypericaceae, Lecythidaceae, Malvaceae, Marsileaceae, Meliaceae, Moraceae, Moringaceae, Myrtaceae, Nelumbonaceae, Opiliaceae, Phyllanthaceae, Piperaceae, Plantaginaceae, and Polygonaceae.

Analysis of plant parts used (Figure 10) revealed that leaves were the most frequently cited (44 citations; 43.14%), consistent with the widespread consumption of leafy vegetables. Fruits followed with 23 citations (22.55%), often including immature or tender fruiting bodies used as vegetables. Inflorescences accounted for 17 citations (16.67%), reflecting traditional consumption of flower parts, while stems were cited in 11 cases (10.78%). Less frequently used parts included roots, rhizomes, and seeds (2 citations each; 1.96%), and whole plants were least reported (1 citation; 0.98%), demonstrating a comprehensive utilization of plant organs in local diets.

### 3.6. Fidelity Level (FL) of Medicinal Edible Plants in MY

The Fidelity Level (FL) analysis provides valuable insights into the degree of cultural consensus regarding the medicinal uses of edible plants. FL quantifies the proportion of informants who cite a species for its primary medicinal purpose, thereby indicating the cultural importance and perceived efficacy of that use within the local healthcare context.

In this study, several species attained the maximum FL value of 100%, signifying complete agreement among informants on their key therapeutic application. Notably, *Centella asiatica* demonstrated unanimous citation for its use in alleviating internal bruising and inflammation related to musculoskeletal conditions. Similarly, *Streptocaulon juventas* showed a high FL of 90.91% for promoting postpartum recovery through expulsion of lochia and impure blood.

High FL values were also recorded in species such as *Euphorbia hirta* (71.43%) for treatment of dysuria and hematuria, *Orthosiphon aristatus* (71.43%) for blood pressure regulation and diabetes management, *Curcuma comosa* (69.23%) for uterine health and alleviating uterine cramps, and *Andrographis paniculata* (68.75%) for treating febrile illnesses. These results suggest strong cultural validation and trust in the effectiveness of these plants for specific conditions.

For *Achyranthes aspera* L., the FL value of 54.55% indicates a moderate degree of cultural consensus. Informants primarily cited the whole plant, used in decoction, for its traditional role in reducing fever. This relatively moderate FL suggests that while *A. aspera* is recognized for its therapeutic value, there is somewhat less agreement on its primary use compared to species with higher FL values. This may reflect the plant’s secondary importance in the community’s healthcare practices or variations in its application across different localities or informants.

Several species exhibited moderate FL values (40–70%), reflecting multifunctional roles or slightly less consensus on their primary use. For example, *Zingiber montanum* had FLs of 63.64% and 36.36% for gynecological and gastrointestinal uses, respectively, while *Blumea balsamifera* showed FLs ranging from 22.22% to 55.56% across fever reduction, antiparasitic, and gastrointestinal indications.

Lower FL values (<40%) were generally observed in species reported with multiple uses, diluting consensus for any one application. For instance, *Hellenia speciosa* was cited for digestive support (57.69%), skin disorders (23.08%), and urinary tract conditions (19.23%). Such versatility reflects the complex traditional knowledge systems but leads to lower FL values due to divided informant agreement.

A detailed summary of species, used parts, preparation methods, therapeutic uses, and corresponding FL values is provided in Table S2.

### 3.7. Informant Consensus Factor (F_ic_) of Medicinal Edible Plants in MY

The Informant Consensus Factor (F_ic_) was calculated to assess the degree of agreement among informants regarding the medicinal use of plant species across different therapeutic categories (Table 2). Reported ailments were grouped into major physiological and ethnomedicinal categories based on previous ethnobotanical studies and the World Health Organization’s International Classification of Diseases (ICD-11) [34]. The categories identified in this study included the following: (1) reproductive system, (2) poisoning and toxicology, (3) musculoskeletal and joint diseases, (4) skin conditions, (5) nutrition and blood-related disorders, (6) gastrointestinal system disorders, (7) obstetrics, gynecology, and urinary tract disorders, and (8) infections, parasites, and immune system disorders.

The F_ic_ values ranged from 0.89 to 1.00, indicating generally high levels of agreement among informants. The highest consensus (F_ic_ = 1.00) was observed in the reproductive system and poisoning and toxicology categories, each with a single reported taxon, suggesting unanimous agreement on the use of one specific plant species in these contexts. High levels of consensus were also found in musculoskeletal and joint diseases (F_ic_ = 0.94), skin conditions (F_ic_ = 0.92), and nutrition and blood-related disorders (F_ic_ = 0.91), indicating that few taxa were frequently cited for these ailments.

The gastrointestinal category had the highest number of use reports (N_ur_ = 130) and maintained a high consensus (F_ic_ = 0.91), suggesting broad use and shared knowledge among informants. Similarly, obstetrics, gynecology, and urinary disorders (F_ic_ = 0.90) and infections, parasites, and immune system disorders (F_ic_ = 0.89) also demonstrated strong agreement, though with slightly more variability in plant selection.

### 3.8. Cultural Food Significance Index (CFSI) of Edible Plants in MY

A total of 170 edible plant species were evaluated for their cultural food significance in MY, using the Cultural Food Significance Index (CFSI) (Tables S3 and S4). The CFSI values showed considerable variation, reflecting differences in frequency of use, availability, versatility of plant parts used, medicinal relevance, and taste appreciation within the local food culture.

The species with the highest CFSI was *Hellenia speciosa*, scoring 3447.60, indicating its exceptional cultural relevance as both a food and medicinal resource. This high value was supported by strong scores across nearly all contributing indices, particularly high availability (AI = 34), frequency of utilization (FUI = 2), parts used (PUI = 3.25), multi-functional food use (MFFI = 1), taste score appreciation (TSAI = 7.5), and a food–medicinal role (FMRI = 4). These values suggest the species plays a significant role in both traditional diets and healthcare practices.

*Allium cepa* ranked second with a CFSI of 2187.00, followed by *Citrus × aurantiifolia* (846.00), *Cymbopogon citratus* (799.20), and *Senna siamea* (792.00). These species demonstrated high frequency of utilization (FUI = 5 for all), good taste appreciation (TSAI ranging from 9 to 10), and frequent food–medicinal use, emphasizing their broad ethnobotanical roles in the region.

Species with moderate CFSI values, such as *Oroxylum indicum* (391.50), *Piper sarmentosum* (349.92), and *Zingiber officinale* (307.80), are regularly consumed as culinary ingredients. In contrast, species such as *Garcinia mangostana* (103.68), *Orthosiphon aristatus* (92.40), and *Telosma cordata* (54.68) recorded lower CFSI values, indicating more limited or specialized cultural roles in local diets.

**Figure 11:**
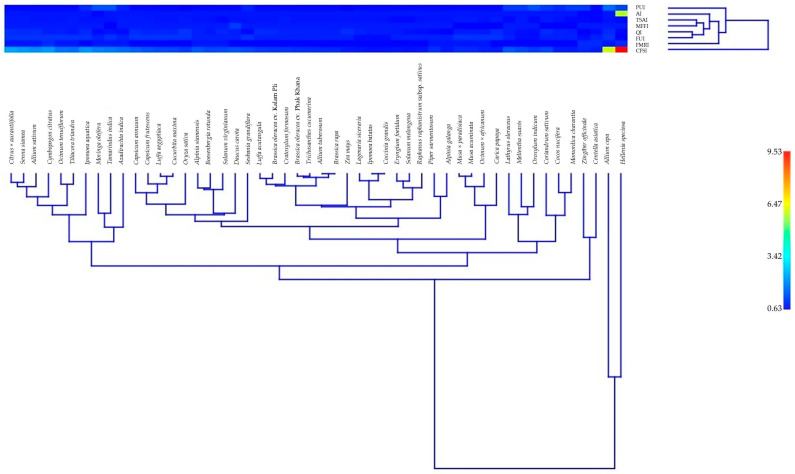
Heatmap analysis of edible plants in MY.

The heatmap analysis (Figure 11) visually demonstrated the variation in CFSI component indices across species. *Andrographis paniculata* and *Barleria prionitis* showed strong clustering due to their consistently high QI, AI, FUI, and FMRI scores, further confirming their central role in local food traditions. Conversely, species such as *Dolichandrone serrulata*, *Polyalthia evecta*, and *Raphanus raphanistrum* subsp. *sativus* were associated with lower cluster positions, reflecting modest or niche usage patterns.

Interestingly, several species with relatively high PUI and MFFI values, such as *Basella alba* and *Ocimum tenuiflorum*, demonstrated versatility in how they are consumed—ranging from cooked dishes to herbal infusions. This flexibility likely contributes to their elevated cultural food significance.

Species with low FMRI scores tended to have minimal association with medicinal uses, which may explain their reduced overall CFSI values despite being edible. However, some species—like *Cryptolepis buchananii* and *Centella asiatica*—showed balanced scores across multiple indices, underscoring their dual role in nutrition and folk medicine.

The integration of these ethnobotanical indices through the CFSI framework provides a comprehensive tool for identifying culturally significant edible plant species. These findings not only reflect local dietary preferences and traditional knowledge but also offer potential candidates for future research, conservation, and sustainable food development initiatives in Northeastern Thailand.

## 4. Discussion

### 4.1. Edible Plant Diversity and Family Composition

The present study identified 170 edible plant species belonging to 60 families in MY, demonstrating a rich ethnobotanical heritage and significant plant biodiversity within this region. The diversity recorded here surpasses several previous surveys conducted in Northeastern Thailand [14,35], suggesting that comprehensive field approaches and inclusion of varied land-use types have captured a broader spectrum of species utilized by local communities [36].

Fabaceae emerged as the most species-rich family (15.88%), followed by Cucurbitaceae (7.06%) and Zingiberaceae (5.88%), reflecting global and regional patterns of ethnobotanical prominence [37]. The high Fabaceae diversity aligns with their ecological versatility and nutritional importance, particularly as protein sources and nitrogen fixers that enhance soil fertility [38]. The presence of 27 families represented by a single species highlights specialized local knowledge and underscores the importance of preserving lesser-known taxa, which may have unique cultural or nutritional roles [39].

### 4.2. Native and Introduced Species: Dynamics of Biocultural Diversity

The nearly equal proportions of native (52.94%) and introduced species (46.47%) illuminate the dynamic nature of local agroecosystems, shaped by historical biogeography, trade, and cultural exchanges. This balance mirrors findings from other ethnobotanical studies in tropical Asia, where introduced species complement indigenous biodiversity to meet evolving dietary and economic needs [40,41]. The integration of introduced species, such as *Zea mays* and *Citrus × aurantiifolia*, reflects adaptive strategies for food security amid environmental and market changes, while native species maintain cultural identity and ecological resilience [42]. Such coexistence supports the concept of biocultural diversity as a driver of sustainability in traditional food systems [43].

### 4.3. Cultivated Versus Wild Edible Plants: Complementary Food Procurement Strategies

The dominance of cultivated species (72.35%) emphasizes the central role of agroecosystems and home gardens in sustaining local diets and livelihoods, consistent with ethnobotanical research in rural Southeast Asia [44]. Nevertheless, the notable presence of wild-harvested species (17.65%) underscores their critical function in dietary diversification, micronutrient supplementation, and cultural practices, echoing broader global findings on the importance of wild foods for rural nutrition and resilience [45].

This dual strategy enables communities to buffer against crop failure, market fluctuations, and seasonal food shortages, reinforcing food sovereignty and ecological stewardship [46]. It also highlights the cultural significance of wild species, which often have medicinal, ritual, or flavor attributes not found in cultivated counterparts [47].

### 4.4. Threats to Edible Plant Species and Their Sustainable Use

While this study provides valuable insights into the diversity and cultural significance of edible plant species in MY, it is important to recognize the potential threats that could jeopardize the future availability of these plants. Specifically, climate change, land-use shifts, and invasive species represent significant challenges that need to be addressed for the continued sustainability of local food systems.

Climate change is expected to bring more extreme weather events, unpredictable rainfall, and temperature fluctuations, which could disrupt the growing conditions of many edible plant species [48]. Crops like *Oryza sativa* and *Zea mays* are particularly vulnerable, as they rely on stable environmental conditions. These changes may lead to lower crop yields or even the loss of certain species that are unable to adapt quickly enough [49]. Shifts in climate could also impact the seasonal availability of wild-harvested plants, further affecting food security in the region [50].

Expanding agricultural activities, deforestation, and urbanization are altering the landscape in MY. As forests and natural habitats are cleared for farming or development, many plant species, both cultivated and wild, may lose their habitats [51]. Furthermore, the increasing trend toward monoculture farming, where only one or two crops are grown extensively, could limit plant diversity and reduce the availability of important edible species. These shifts in land use can negatively affect both the biodiversity of local ecosystems and the richness of local diets [52].

The introduction of non-native species, both intentionally and accidentally, can disrupt local ecosystems. Invasive plants may outcompete native edible species, reducing the availability of these plants in the wild or even in cultivated areas [53]. The spread of invasive plant species can also undermine traditional agricultural practices, making it harder for local communities to maintain their food systems [54].

### 4.5. Species Use Value (SUV) and Cultural Significance

The Species Use Value (SUV) provides a clear indication of the cultural and functional importance of edible plants in the local community. The highest SUV was recorded for *Oryza sativa* (1.00), emphasizing its foundational role as the primary staple food and a cultural keystone species in the region. This underscores the centrality of rice in the local diet and its critical significance in daily life [55]. Close behind, *Zea mays* (0.96) and *Citrus × aurantiifolia* (0.94) also hold substantial importance, reflecting their nutritional, culinary, and economic value in the community. Both species are integral to the local culinary system, often grown in household gardens and used in various traditional dishes and beverages [37]. These findings align with ethnobotanical studies in Thailand and neighboring Southeast Asian countries, where staple grains and widely cultivated fruits consistently dominate SUV rankings [56].

Interestingly, species like *Allium sativum* and *Coriandrum sativum* exhibited relatively high use intensity with SUV values of 0.92 and 0.90, despite their moderate citation frequencies. This suggests that these plants play specialized culinary and medicinal roles, valued for their distinct contributions to food preparation and traditional medicine [57]. The relatively high SUV for these plants highlights the duality of ethnobotanical indices, capturing both widespread and niche uses of plants in the local culture, thus providing a deeper understanding of their significance [58].

While the Species Use Value (SUV) and associated ethnobotanical indices provide valuable insights into the cultural and functional importance of edible plants, this study did not include a systematic assessment of their economic significance in local markets. Specifically, data on the frequency, volume, and pricing of these species in community trade or regional commerce were not collected. We recognize this as a limitation, as market value plays a crucial role in shaping both the practical and perceived value of plant species. Future research should incorporate quantitative market surveys and livelihood assessments to better capture the economic contributions of key edible plants to local households and economies.

### 4.6. Family Use Value (FUV) and Functional Importance

Families with the highest FUV—Caricaceae (0.900), Amaryllidaceae (0.860), and Anacardiaceae (0.840)—demonstrate disproportionate importance relative to species richness, suggesting that certain families possess highly valued species integral to local food and health systems. This pattern corroborates other studies emphasizing the role of key plant families in traditional diets and ethnomedicine [59,60]. Conversely, Fabaceae, despite being the most species-rich family, had a moderate FUV (0.601), possibly due to the wide variation in use intensity across its many species [35].

These disparities reflect complex socio-ecological interactions, including cultural preferences, availability, and historical usage patterns, emphasizing the need for family-level analyses to complement species-level assessments in ethnobotanical research.

### 4.7. Relative Frequency of Citation (RFC) and Its Relationship with Use Intensity

The strong positive correlation between RFC and SUV for most species supports the effectiveness of RFC as an indicator of cultural prominence and community consensus regarding the medicinal or culinary importance of the species [25]. However, there are notable exceptions: some species with high citation rates demonstrate moderate use intensity, which may reflect symbolic, ecological, or ritual significance rather than regular consumption or medicinal use. Conversely, certain species with moderate citation frequencies exhibit concentrated but intense uses, often in specific contexts or for particular therapeutic applications [61].

These findings highlight the multidimensional nature of plant significance in traditional knowledge systems. It underscores the importance of integrating multiple quantitative ethnobotanical indices to fully capture both the breadth of plant use (how widely a species is cited) and the depth of its use (the intensity and frequency of its application). This approach provides a more holistic understanding of the cultural and functional roles of plants within local communities [62].

### 4.8. Diversity and Multifunctional Uses of Edible Plants in Local Food Systems

This ethnobotanical study provides a comprehensive documentation and analysis of edible plant utilization across multiple domains in MY, revealing a rich and diverse biocultural heritage. The identification of 23 species used as beverages, 24 as condiments and flavoring, and 90 as vegetables, among other categories, underscores the integral role of wild and cultivated plants in sustaining local food systems and cultural identity.

The predominance of fruits in beverage preparation (69.57%) aligns with patterns observed in other tropical Asian communities, where fruit juices, fermented drinks, and decoctions form a central part of traditional diets [63,64]. The emphasis on Apiaceae, Musaceae, and Poaceae families in beverage use highlights the intersection of ecological availability and cultural preferences, with these families providing both nutritional and phytochemical benefits [65,66,67]. The frequent use of leaves in condiments (36%) reflects a well-documented ethnobotanical trend where aromatic foliage contributes complex flavors and medicinal properties, as seen in related studies across Thailand and neighboring countries [37,68].

Fermentation and preservation practices documented here, particularly the prominent role of Poaceae species, demonstrate an enduring reliance on rice and cereal grains, not only as staples but also as bases for value-added fermented foods [69]. Such practices are critical for food security, extending shelf life and enhancing nutritional profiles in seasonal and resource-variable contexts [70]. The use of stems and leaves in these processes reflects adaptive strategies that maximize plant parts beyond primary consumption [71].

The documentation of 42 fruit species from 26 families, with Moraceae, Annonaceae, and Phyllanthaceae most represented, attests to the region’s floristic richness and the community’s sophisticated use of seasonal and perennial fruiting plants [72,73]. This diversity supports dietary diversity and micronutrient intake, which are vital for nutritional health in rural populations [74]. The exclusive use of fruits in this category emphasizes their role as a primary edible part, consumed fresh or processed [75].

Medicinal edible plants hold a significant dual function, reinforcing the deep integration of nutrition and traditional healthcare systems in the community [76]. The high-Fidelity Level (FL) values, particularly for *Centella asiatica* and *Streptocaulon juventas*, demonstrate strong cultural consensus on the efficacy of these species, consistent with pharmacological evidence supporting their anti-inflammatory and postpartum recovery properties [77,78]. The predominance of leaves in medicinal use (25%) suggests both ease of sustainable harvest and broad therapeutic utility, corroborating findings in ethnopharmacology that highlight foliage as a principal source of bioactive compounds, such as madecassic acid, madasiatic acid, and asiatic acid [79].

Staple foods, dominated by *Oryza sativa* and *Zea mays*, reflect global dietary patterns where cereals underpin energy intake and cultural food identities [80,81]. Their exclusive use of seeds as reported confirms their central nutritional role and the versatility of processing methods employed locally. This underscores the continued importance of traditional crop varieties in food sovereignty amidst modern agricultural changes [82].

The diversity of species used in sweets and snacks, with Fabaceae as a dominant family, reveals an interesting dimension of plant utilization often underrepresented in ethnobotanical literature [83]. The varied plant parts used, from fruits to seeds and roots, highlight multifunctionality and innovation in local culinary traditions [1]. The broad spectrum of edible vegetables (90 species) spanning 36 families points to an extensive knowledge system managing wild and cultivated resources, crucial for dietary diversity and resilience [37].

### 4.9. Fidelity Level (FL): Cultural Agreement and Therapeutic Focus

The high FL values observed—particularly for *Centella asiatica* (FL = 100%) and *Streptocaulon juventas* (FL = 90.91%)—reflect a strong consensus on their specific medicinal applications [84]. This is consistent with studies conducted in other parts of Thailand and Southeast Asia, where *Centella asiatica* is widely recognized for its anti-inflammatory and wound-healing properties [85]. The high FL value for *Streptocaulon juventas* as a postpartum tonic reflects its prominent role in culturally significant postpartum care practices involving herbal medicine [14].

Conversely, plants with multifunctional uses, such as *Hellenia speciosa* and *Blumea balsamifera*, exhibited lower FL values, likely because informant responses were distributed across multiple therapeutic uses, reducing consensus for any single application [86]. Similarly, *Achyranthes aspera* (FL = 54.55%) demonstrated a moderate degree of consensus, primarily being cited for its traditional use in fever reduction. The moderate FL value for *Achyranthes aspera* suggests that while it is recognized for its medicinal value, there is less agreement on its primary use compared to species with higher FL values. This may reflect its secondary importance in the community’s healthcare practices or variations in its application across different informants and localities.

### 4.10. Informant Consensus Factor (F_ic_): Validation of Therapeutic Categories

The high F_ic_ values across most therapeutic categories (0.89–1.00) reflect a shared understanding and transmission of ethnomedicinal knowledge within the community [87]. Particularly noteworthy are the reproductive system and toxicology categories (F_ic_ = 1.00), where single-species usage indicates deeply rooted cultural practices with minimal variation, reflecting strong consensus among informants in these critical health domains [88].

The gastrointestinal system, with the highest number of use reports (N_ur_ = 130) and a high F_ic_ (0.91), mirrors trends seen in other rural regions where digestive health is a common focus of traditional medicine [89]. This reinforces the importance of plant-based treatments for everyday ailments and suggests strong oral transmission of knowledge related to dietary-medicinal overlap [90].

### 4.11. Cultural Food Significance Index (CFSI): Food-Medicine Continuum

The application of the Cultural Food Significance Index (CFSI) provided a comprehensive understanding of the cultural relevance of plants, revealing layers of significance that go beyond mere usage frequency. For instance, *Hellenia speciosa*, which received the highest CFSI value of 3447.60, exemplifies how a plant’s significance extends beyond its medicinal properties to include its culinary versatility and availability. This mirrors how abundant, multifunctional species often become dietary staples in indigenous food systems, where their medicinal and nutritional roles are deeply intertwined [1].

Species such as *Allium cepa*, *Cymbopogon citratus*, and *Citrus* × *aurantiifolia* ranked highly in the CFSI due to their combined appeal in taste and their dual food–medicinal uses. These plants illustrate the concept of functional foods, highlighting the intersection of culinary enjoyment and health benefits [91]. In contrast, plants like *Telosma cordata* and *Orthosiphon aristatus*, despite being edible, showed lower CFSI values due to their more specialized roles, both in food and medicine. This suggests that while these species may play important roles in local health systems, their culinary significance remains limited [92].

Additionally, heatmap clustering revealed patterns of use that resonate with cultural specialization in plant selection. *Andrographis paniculata* and *Barleria prionitis* emerged as dual-purpose plants, valued for both their medicinal and culinary applications, while species like *Polyalthia evecta* demonstrated more specialized and niche uses [93,94,95]. This diversity reflects adaptive strategies within local food systems, where communities balance multifunctionality and specialization to optimize health, nutrition, and cultural traditions [14,35].

### 4.12. Contributions to Ethnobotanical Theory and Conservation Practice

Collectively, the high levels of agreement reflected in FL and F_ic_, alongside detailed CFSI analyses, underscore the rich ethnobotanical heritage of Yasothon Province. These findings not only align with prior regional studies but also offer new contributions by quantifying the food–medicine interface in a culturally specific context. The integration of quantitative indices in this study strengthens the case for preserving both biodiversity and associated indigenous knowledge systems.

Furthermore, the identification of species with both high cultural significance and medicinal efficacy, such as *Centella asiatica*, *Cymbopogon citratus*, and *Hellenia speciosa*, highlights candidates for further pharmacological and agronomic research. These plants have potential in sustainable food system development, especially within the context of climate change and local health sovereignty.

### 4.13. Summary of Main Findings

This study highlights the extensive diversity of edible plants in Yasothon Province, documenting 170 species across 60 families, which underscores the community’s rich ethnobotanical heritage and adaptive food system. Cultivated species (72.35%) form the backbone of local diets, while wild plants (17.65%) continue to play critical roles in nutrition, medicine, and cultural practices. Native and introduced species are nearly equally represented (52.94% and 46.47%, respectively), illustrating the dynamic integration of traditional and introduced crops. Quantitative indices provided deeper insights into species significance. *Oryza sativa* exhibited the highest Species Use Value (SUV = 1.00), reaffirming its central role as a staple food and cultural keystone. Other species with high SUV values include *Zea mays* (0.96), *Citrus × aurantiifolia* (0.94), *Allium sativum* (0.92), and *Coriandrum sativum* (0.90), reflecting their multifunctional use in food, medicine, and rituals. High Fidelity Level (FL) values for *Centella asiatica* (FL = 100%) and *Streptocaulon juventas* (FL = 90.91%) indicate strong agreement on their specific medicinal uses, particularly for inflammation and postpartum care. Informant Consensus Factor (F_ic_) values ranged from 0.89 to 1.00, with reproductive and gastrointestinal categories showing especially high consensus, validating the reliability of ethnomedicinal knowledge. The Cultural Food Significance Index (CFSI) further emphasized the multifunctionality of plants like *Hellenia speciosa* (CFSI = 3447.60), *Allium cepa* (CFSI = 2187.00), and *Cymbopogon citratus* (CFSI = 799.20), which are valued for both culinary and therapeutic uses. Together, these findings demonstrate a well-integrated food–medicine continuum and highlight the importance of preserving both plant biodiversity and traditional ecological knowledge in the face of environmental and socioeconomic changes.

## 5. Conclusions

This study documents a rich diversity of 170 edible plant species from 60 families in MY, reflecting a vibrant ethnobotanical heritage and complex biocultural relationships. The balanced presence of native and introduced species, alongside both cultivated and wild-harvested plants, underscores adaptive strategies that sustain local food security, nutrition, and cultural identity.

Quantitative analyses reveal that staple cereals such as *Oryza sativa* and *Zea mays* remain central to local diets, while multifunctional species like *Hellenia speciosa* and *Cymbopogon citratus* demonstrate the intricate integration of food and medicine through the food–medicine continuum. High fidelity and consensus indices highlight strong cultural agreement on the therapeutic roles of key species, affirming the deep interconnection between traditional knowledge and health practices.

The diversity of plant uses—from beverages and condiments to sweets and medicinal foods—illustrates a holistic approach to food systems that embraces both multifunctionality and specialization. This complexity not only supports dietary diversity and resilience but also provides valuable insights for biodiversity conservation and sustainable development.

Ultimately, this ethnobotanical investigation emphasizes the critical importance of preserving both plant biodiversity and indigenous knowledge to enhance food sovereignty and community well-being amid changing environmental and socioeconomic conditions. Building on these findings, future research could explore the nutritional and medicinal properties of key species, sustainable cultivation and harvesting practices, and community-based programs to transmit traditional knowledge to younger generations, thereby ensuring the continuity of ethnobotanical heritage and contributing to resilient, locally adapted food and health systems.

While this study does not include experimental validation, future research could focus on phytochemical analysis to identify active compounds and pharmacological studies to assess their biological activities. Such research would provide the scientific foundation needed to support or challenge the medicinal claims made by traditional healers, further integrating traditional knowledge with modern science and fostering sustainable health solutions.

## Figures and Tables

**Table 1:** Family-level, number of species, and Family Use Value (FUV) of edible plants documented in MY.

Family	Number of Species	FUV	Family	Number of Species	FUV	Family	Number of Species	FUV
Caricaceae	1	0.900	Bromeliaceae	1	0.600	Malvaceae	4	0.490
Amaryllidaceae	3	0.860	Elaeocarpaceae	1	0.600	Irvingiaceae	1	0.480
Anacardiaceae	2	0.840	Lamiaceae	4	0.580	Nelumbonaceae	1	0.480
Alismataceae	1	0.800	Arecaceae	3	0.567	Sapotaceae	2	0.480
Convolvulaceae	2	0.800	Basellaceae	1	0.560	Zingiberaceae	10	0.480
Hypericaceae	1	0.780	Bignoniaceae	2	0.560	Moraceae	4	0.460
Cucurbitaceae	12	0.767	Lythraceae	1	0.560	Asteraceae	3	0.440
Solanaceae	6	0.747	Moringaceae	1	0.560	Plantaginaceae	1	0.440
Apiaceae	7	0.734	Meliaceae	3	0.547	Apocynaceae	5	0.384
Musaceae	2	0.730	Araceae	3	0.547	Muntingiaceae	1	0.380
Poaceae	8	0.718	Oxalidaceae	1	0.540	Rhamnaceae	2	0.370
Brassicaceae	6	0.703	Polygonaceae	1	0.540	Passifloraceae	1	0.360
Sapindaceae	2	0.699	Amaranthaceae	3	0.527	Phyllanthaceae	5	0.336
Rutaceae	4	0.695	Costaceae	1	0.520	Celastraceae	1	0.320
Cleomaceae	1	0.680	Marsileaceae	1	0.520	Rubiaceae	2	0.300
Menispermaceae	1	0.680	Nymphaeaceae	2	0.520	Acanthaceae	3	0.287
Clusiaceae	1	0.640	Pandanaceae	1	0.520	Colchicaceae	1	0.280
Myrtaceae	3	0.633	Piperaceae	2	0.520	Euphorbiaceae	2	0.260
Opiliaceae	1	0.620	Annonaceae	3	0.500	Vitaceae	1	0.220
Fabaceae	19	0.601	Lecythidaceae	1	0.500	Connaraceae	1	0.200

**Table 2:** Informant consensus factor (F_ic_) of edible plants in MY.

Therapeutic Categories	Number of Use Report (N_ur_)	Number of Taxa (N_t_)	F_ic_
Reproductive System	8	1	1.00
Poisoning and Toxicology	9	1	1.00
Musculoskeletal and Joint Diseases	63	5	0.94
Skin System	14	2	0.92
Nutrition and Blood	23	3	0.91
Gastrointestinal	130	13	0.91
Obstetrics, Gynaecology, and Urinary Disorders	59	7	0.90
Infection, Parasite, and Immune System	67	8	0.89

## Data Availability

The data presented in this study are available on request from the corresponding authors.

## Supplementary Materials

The following supporting information can be downloaded at: https://www.mdpi.com/article/10.3390/biology14091264/s1, Table S1. Edible plant species documented in Mueang District, Yasothon Province, Thailand. The table includes data on each species’ botanical family, scientific and vernacular names, distribution status, resource origin, used parts, mode of utilization, Species Use Value (SUV), Relative Frequency of Citation (RFC), Cultural Food Significance Index (CFSI), and voucher specimen numbers. Table S2: Fidelity Level (FL) evaluation of edible plants in Mueang District, Yasothon Province. Table S3: Cultural Food Significance Index (CFSI) evaluation of edible plants in Mueang District, Yasothon Province. Table S4: Analysis and calculation of the Cultural Food Significance Index (CFSI).
