# Supplementary material for: Ethnobotanical Heritage of Edible Plants Species in Mueang District, Yasothon Province, Northeastern Thailand
Source: Biology (Basel). 2025 Sep 13;14(9):1264. doi: 10.3390/biology14091264 (PMC12467265; doi:10.3390/biology14091264)
Supplement: Supplementary file 1 [file biology-14-01264-s001.zip › biology-3799442-supplementary.pdf]

biology

MDPI

Supplementary

Ethnobotanical Heritage of Edible Plants in Mueang District, Yasothon Province, North-eastern Thailand

Supplementary

Table S1. Edible Plant Species Documented in Mueang District, Yasothon Province. Thailand. The table includes data on each species' botanical family, scientific and vernacular names, distri-bution status, resource origin, used parts, mode of utilization, Species Use Value (SUV), Relative Frequency of Citation (RFC), Cultural Food Significance Index (CFSI), and voucher specimen numbers.

No.	Family	Scientific name	Vernacular name	Distribution	Resource	Used parts	Utilization	SUV	RFC	CFSI	Voucher no.
1.	Acanthaceae	*Andrographis panicu-lata* (Burm.f.) Wall. ex Nees	Fa Thalai Chon	Introduced	C	LV, WP	MP	0.32	0.30	79.20	TJY0069
2.	Acanthaceae	*Barleria prionitis* L.	Angkap Nu	Introduced	C	WP	MP	0.24	0.24	39.60	TJY0070
3.	Acanthaceae	*Thunbergia laurifolia* Lindl.	Rang Chuet	Introduced	C	LV	MP	0.30	0.28	58.50	TJY0071
4.	Alismataceae	*Limnocharis flava* (L.) Buchenau	Phak Kan Chong	Introduced	B	IF	VG	0.80	0.78	129.60	TJY0091
5.	Amaranthaceae	*Achyranthes aspera* L.	Phan Ngu	Native	W	RT, WP	MP	0.22	0.22	54.45	TJY0092
6.	Amaranthaceae	*Amaranthus blitum* L.	Phak Khom	Introduced	C	LV	VG	0.70	0.72	141.75	TJY0093
7.	Amaranthaceae	*Amaranthus viridis* L.	Phak Khom Hat	Introduced	C	LV	VG	0.66	0.70	133.65	TJY0094
8.	Amaryllidaceae	*Allium cepa* L.	Hom Daeng	Introduced	C	BU, WP	FP, CF, VG	0.90	0.84	2187.00	TJY0095
9.	Amaryllidaceae	*Allium sativum* L.	Krathiam	Introduced	C	BU	CF	0.92	0.88	745.20	TJY0096
10.	Amaryllidaceae	*Allium tuberosum* Rottler ex Spreng.	Phak Paen	Introduced	C	LV	FP, VG	0.76	0.70	328.32	TJY0097
11.	Anacardiaceae	*Mangifera indica* L.	Mamuang	Native	C	FT	BV, FR, SW	0.90	0.88	162.00	TJY0103
12.	Anacardiaceae	*Spondias pinnata* (L.f.) Kurz	Makok	Native	C	FT	CF	0.78	0.70	210.60	TJY0104
13.	Annonaceae	*Annona squamosa* L.	Noina	Introduced	C	FT	FR	0.84	0.80	136.08	TJY0004

14.	Annonaceae	*Polyalthia debilis* (Pierre) Finet & Gagnep.	Kluai Tao	Native	W	FT	FR	0.38	0.32	7.41	TJY0031
15.	Annonaceae	*Polyalthia evecta* (Pierre) Finet & Gagnep.	Nom Noi	Native	W	FT	FR	0.28	0.32	5.46	TJY0032
16.	Apiaceae	*Anethum graveolens* L.	Phak Chi-lao	Introduced	C	LV	VG	0.80	0.84	172.80	TJY0072
17.	Apiaceae	*Apium sellowianum* H.Wolff	Phak Khuen Chai	Introduced	C	LV	VG	0.84	0.80	181.44	TJY0073
18.	Apiaceae	*Centella asiatica* (L.) Urb.	Buabok	Native	C	LV	BV, MP	0.58	0.52	271.44	TJY0074
19.	Apiaceae	*Coriandrum sativum* L.	Phakchi	Introduced	C	LV, RT	CF, VG	0.90	0.80	432.00	TJY0075
20.	Apiaceae	*Daucus carota* L.	Khaerot	Introduced	C	RT	BV, VG	0.76	0.62	492.48	TJY0076
21.	Apiaceae	*Eryngium foetidum* L.	Hom Phe	Introduced	C	LV	VG	0.70	0.60	252.00	TJY0077
22.	Apiaceae	*Oenanthe javanica* (Blume) DC.	Phak Chi-lom	Native	C	LV	VG	0.56	0.56	32.76	TJY0078
23.	Apocynaceae	*Carissa carandas* L.	Manao Ho	Introduced	W	FT	FR	0.56	0.52	21.84	TJY0162
24.	Apocynaceae	*Cryptolepis buchananii* R.Br. ex Roem. & Schult.	Thao En On	Native	W	LV, VN	MP	0.32	0.34	22.00	TJY0163
25.	Apocynaceae	*Myriopteron extensum* (Wight) K.Schum.	Cha Em	Native	C	SM	CF	0.28	0.30	21.84	TJY0164
26.	Apocynaceae	*Streptocaulon juventas* (Lour.) Merr.	Khreau Prasong	Native	W	RT, WP	MP	0.22	0.32	27.23	TJY0165
27.	Apocynaceae	*Telosma cordata* (Burm.f.) Merr.	Khachon	Native	C	IF	VG	0.54	0.48	54.68	TJY0166
28.	Araceae	*Amorphophallus brevispathus* Gagnep.	Elok	Native	W	SM	VG	0.50	0.44	54.00	TJY0178
29.	Araceae	*Colocasia esculenta* (L.) Schott	Bon	Native	W	SM	VG	0.44	0.44	34.32	TJY0179
30.	Araceae	*Wolffia globosa* (Roxb.) Hartog & Plas	Phum	Native	B	LV	VG	0.70	0.68	126.00	TJY0180
31.	Arecaceae	*Calamus viminalis* Willd.	Wai Khom	Native	W	SM	VG	0.42	0.52	33.60	TJY0187
32.	Arecaceae	*Cocos nucifera* L.	Maphrao	Introduced	C	FT, SM	BV, FR, VG	0.78	0.76	280.80	TJY0188
33.	Arecaceae	*Salacca wallichiana* Mart.	Rakam	Native	C	FT	FR	0.50	0.48	40.50	TJY0189

34.	Asteraceae	*Blumea balsamifera* (L.) DC.	Nat Yai	Native	W	LV	MP	0.18	0.20	7.43	TJY0193
35.	Asteraceae	*Lactuca sativa* L.	Phak Kat Hom	Introduced	C	LV	VG	0.82	0.78	166.05	TJY0194
36.	Asteraceae	*Pluchea indica* (L.) Less.	Khlu	Native	C	LV, WP	MP, VG	0.32	0.30	126.72	TJY0195
37.	Basellaceae	*Basella alba* L.	Phak Plang	Native	C	IF	VG	0.56	0.50	56.70	TJY0175
38.	Bignoniaceae	*Dolichandrone serrulata* (Wall. ex DC.) Seem.	Khae Na	Native	C	IF	VG	0.54	0.46	72.90	TJY0170
39.	Bignoniaceae	*Oroxylum indicum* (L.) Kurz	Phe Ka	Native	C	FT, IF, LV	VG	0.58	0.56	391.50	TJY0171
40.	Brassicaceae	× *Brassarda juncea* (L.) Su Liu & Z.H.Feng	Phak Kat Hin	Introduced	C	LV	VG	0.62	0.62	111.60	TJY0156
41.	Brassicaceae	*Brassica juncea* (L.) Czern.	Phak Kat	Introduced	C	LV	VG	0.60	0.70	194.40	TJY0157
42.	Brassicaceae	*Brassica oleracea* L. cv. "Phak Khana"	Phak Khana	Introduced	W	LV	VG	0.80	0.76	345.60	TJY0158
43.	Brassicaceae	*Brassica oleracea* L. cv. "Kalam Pli"	Kalam Pli	Introduced	C	LV	FP, VG	0.86	0.78	371.52	TJY0159
44.	Brassicaceae	*Brassica rapa* L.	Phak Kwangtung	Introduced	C	LV	VG	0.76	0.74	328.32	TJY0160
45.	Brassicaceae	*Raphanus raphanistrum* subsp. *sativus* (L.) Domin	Hua Chai Thao	Introduced	C	RT	VG	0.58	0.64	250.56	TJY0161
46.	Bromeliaceae	*Ananas comosus* (L.) Merr.	Sapparot	Introduced	C	FT	BV, FR	0.60	0.66	97.20	TJY0173
47.	Caricaceae	*Carica papaya* L.	Malako	Introduced	C	FT	FR	0.90	0.88	270.00	TJY0182
48.	Celastraceae	*Salacia chinensis* L.	Kam-phaeng Chet Chan	Native	W	SM	MP	0.32	0.32	35.20	TJY0190
49.	Cleomaceae	*Cleome gynandra* L.	Phak sian	Native	C	WP	FP	0.68	0.60	110.16	TJY0191
50.	Clusiaceae	*Garcinia mangostana* L.	Mangkhut	Introduced	B	FT	BV, FR	0.64	0.60	103.68	TJY0196
51.	Colchicaceae	*Gloriosa simplex* L.	Dong du-eng	Introduced	C	RT	MP	0.28	0.28	11.55	TJY0200
52.	Connaraceae	*Ellipanthus tomentosus* Kurz	Kham rok	Native	W	SM	MP	0.20	0.18	5.50	TJY0192
53.	Convolvulaceae	*Ipomoea aquatica* Forssk.	Phakbung	Native	C	LV, SM	VG	0.86	0.84	774.00	TJY0176

54.	Convolvulaceae	*Ipomoea batatas* (L.) Lam.	Manthet	Introduced	C	RT	SW	0.74	0.70	239.76	TJY0177
55.	Costaceae	*Hellenia speciosa* (J.Koenig) S.R.Dutta	Ueang Maina	Native	C	IF, RT, SM	MP, VG	0.52	0.48	3447.60	TJY0172
56.	Cucurbitaceae	*Benincasa hispida* (Thunb.) Cogn.	Fak	Introduced	C	FT	VG	0.66	0.66	178.20	TJY0130
57.	Cucurbitaceae	*Citrullus lanatus* (Thunb.) Matsum. & Nakai	Taeng Mo	Introduced	C	FT	BV, FR	0.78	0.70	126.36	TJY0131
58.	Cucurbitaceae	*Coccinia grandis* (L.) Voigt	Tamlueng	Native	C	LV	VG	0.74	0.70	239.76	TJY0132
59.	Cucurbitaceae	*Cucumis melo* L.	Taeng Thai	Introduced	C	FT	SW	0.72	0.68	116.64	TJY0133
60.	Cucurbitaceae	*Cucumis sativus* L.	Taeng Kwa	Native	C	FT	VG	0.84	0.82	181.44	TJY0134
61.	Cucurbitaceae	*Cucurbita maxima* Duchesne	Fakthong	Introduced	C	FT	SW, VG	0.82	0.80	442.80	TJY0135
62.	Cucurbitaceae	*Lagenaria siceraria* (Molina) Standl.	Nam Tao	Introduced	C	FT	VG	0.80	0.84	259.20	TJY0136
63.	Cucurbitaceae	*Luffa acutangula* (L.) Roxb.	Buap Liam	Introduced	C	FT	VG	0.90	0.86	388.80	TJY0137
64.	Cucurbitaceae	*Luffa aegyptiaca* Mill.	Buap Hom	Introduced	C	FT	VG	0.84	0.80	453.60	TJY0138
65.	Cucurbitaceae	*Momordica charantia* L.	Mara Khi Nok	Native	C	FT, LV	VG	0.76	0.72	273.60	TJY0139
66.	Cucurbitaceae	*Sicyos edulis* Jacq.	Fak Maeo	Introduced	C	FT	VG	0.56	0.52	181.44	TJY0140
67.	Cucurbitaceae	*Trichosanthes cucumerina* L.	Buap Ngu	Native	C	FT	VG	0.78	0.68	336.96	TJY0141
68.	Elaeocarpaceae	*Elaeocarpus hygrophilus* Kurz	Makok Nam	Native	C	FT	FR	0.60	0.60	64.80	TJY0167
69.	Euphorbiaceae	*Euphorbia hirta* L.	Namnom Ratchasi	Introduced	W	SM	MP	0.28	0.30	7.70	TJY0168
70.	Euphorbiaceae	*Suregada multiflora* (A.Juss.) Baill.	Khan Thong Phayabat	Native	C	BK	MP	0.24	0.24	6.60	TJY0169
71.	Fabaceae	*Arachis hypogaea* L.	Thua Li-song	Introduced	C	SE	SW	0.72	0.70	207.36	TJY0050
72.	Fabaceae	*Butea monosperma* (Lam.) Kuntze	Charn	Native	C	IF	VG	0.56	0.48	37.80	TJY0051
73.	Fabaceae	*Cassia fistula* L.	Khun	Doubtful	B	IF	VG	0.44	0.44	38.61	TJY0052

74.	Fabaceae	*Clitoria ternatea* L.	Anchan	Introduced	C	IF	BV, VG	0.68	0.60	45.90	TJY0053
75.	Fabaceae	*Lathyrus oleraceus* Lam.	Thua Lantao	Introduced	C	FT, LV	VG	0.72	0.70	518.40	TJY0054
76.	Fabaceae	*Leucaena leucocephala* (Lam.) de Wit	Krathin	Introduced	C	FT, LV	VG	0.64	0.58	172.80	TJY0055
77.	Fabaceae	*Neptunia oleracea* Lour.	Phak Krachet Nam	Native	B	LV	VG	0.70	0.68	189.00	TJY0056
78.	Fabaceae	*Pachyrhizus erosus* (L.) Urb.	Man Kaeo	Introduced	C	CM	SW	0.58	0.52	93.96	TJY0057
79.	Fabaceae	*Parkia speciosa* Hassk.	Sato	Native	C	SE	VG	0.44	0.40	17.16	TJY0058
80.	Fabaceae	*Pithecellobium* dulce (Roxb.) Benth.	Mak Khamthet	Introduced	C	FT	FR	0.54	0.52	31.59	TJY0059
81.	Fabaceae	*Psophocarpus tetragonolobus* (L.) DC.	Thua Phu	Introduced	B	FT	VG	0.78	0.68	168.48	TJY0060
82.	Fabaceae	*Senegalia pennata* (L.) Maslin	Cha Om	Native	C	LV	VG	0.56	0.50	151.20	TJY0061
83.	Fabaceae	*Senna siamea* (Lam.) H.S.Irwin & Barneby	Khilek	Native	C	LV	VG	0.88	0.84	792.00	TJY0062
84.	Fabaceae	*Sesbania grandiflora* (L.) Poir.	Khae	Introduced	C	IF, LV	VG	0.54	0.52	437.40	TJY0063
85.	Fabaceae	*Sesbania javanica* Miq.	Sano	Native	C	IF	VG	0.38	0.40	59.28	TJY0064
86.	Fabaceae	*Tamarindus indica* L.	Makham	Introduced	C	FT, IF, LV	CF, FR, SW	0.64	0.64	648.00	TJY0065
87.	Fabaceae	*Vigna mungo* (L.) Hepper	Thua Dam	Introduced	C	SE	SW	0.58	0.58	90.48	TJY0066
88.	Fabaceae	*Vigna radiata* (L.) R.Wilczek	Thuakhiao	Native	C	SE	SW, VG	0.48	0.52	99.84	TJY0067
89.	Fabaceae	*Vigna unguiculata* subsp. *sesqui-pedalis* (L.) Verdc.	Thuafakyao	Introduced	C	SE	VG	0.56	0.54	80.64	TJY0068
90.	Hypericaceae	*Cratoxylum formosum* (Jack) Benth. & Hook.f. ex Dyer	Phak Tio	Native	B	FT	VG	0.78	0.76	374.40	TJY0174
91.	Irvingiaceae	*Irvingia malayana* Oliv. ex A.W.Benn.	Bok	Native	W	FT	FR	0.48	0.44	28.80	TJY0181
92.	Lamiaceae	*Melissa officinalis* L.	Saranae	Introduced	C	LV	VG	0.56	0.50	226.80	TJY0202
93.	Lamiaceae	*Ocimum × africanum* Lour.	Horapha	Native	C	LV	VG	0.70	0.60	283.50	TJY0203

94.	Lamiaceae	*Ocimum tenuiflorum* L.	Kaphrao	Native	C	LV	CF, VG	0.78	0.70	631.80	TJY0204
95.	Lamiaceae	*Orthosiphon aristatus* (Blume) Miq.	Ya Nuat Maeo	Native	C	FT, LV	MP	0.28	0.30	92.40	TJY0205
96.	Lecythidaceae	*Careya arborea* Roxb.	Phak Kra Don	Native	C	LV	VG	0.50	0.46	60.75	TJY0126
97.	Lythraceae	*Punica granatum* L.	Mak Pila	Introduced	C	FT	BV, FR	0.56	0.50	56.70	TJY0155
98.	Malvaceae	*Abelmoschus esculentus* (L.) Moench	Krachiap Khiao	Introduced	C	FT	VG	0.44	0.44	38.61	TJY0183
99.	Malvaceae	*Bombax anceps* Pierre	Ngio pa	Native	C	IF	CF	0.52	0.48	30.42	TJY0184
100.	Malvaceae	*Cullenia ceylanica* (Gardner) Wight ex K.Schum.	Thurian	Introduced	C	FT	FR, SW	0.70	0.66	94.50	TJY0185
101.	Malvaceae	*Mansonia gagei* J.R.Drumm.	Chanhom	Native	C	SM	MP	0.30	0.20	8.25	TJY0186
102.	Marsileaceae	*Marsilea crenata* C.Presl	Phak Waen	Native	W	LV	VG	0.52	0.48	30.42	TJY0147
103.	Meliaceae	*Azadirachta indica* A.Juss.	Phak Sadao	Native	C	IF, LV	VG	0.56	0.50	604.80	TJY0125
104.	Meliaceae	*Epicharis parasitica* (Osbeck) Mabb.	Langsat	Introduced	C	FT	FR	0.50	0.46	60.75	TJY0198
105.	Meliaceae	*Lansium domesticum* Corrêa	Long Kong	Native	W	FT	FR	0.58	0.48	70.47	TJY0199
106.	Menisperma-ceae	*Tiliacora triandra* (Colebr.) Diels	Yanang	Native	C	LV	CF	0.68	0.66	612.00	TJY0144
107.	Moraceae	*Artocarpus heterophyllus* Lam.	Khanun	Introduced	C	FT	FR, VG	0.72	0.68	155.52	TJY0105
108.	Moraceae	*Ficus racemosa* L.	Maduea Pa	Native	W	FT	FR	0.40	0.40	7.80	TJY0106
109.	Moraceae	*Morus alba* L.	Mon	Introduced	C	FT	FR	0.38	0.44	25.65	TJY0107
110.	Moraceae	*Streblus asper* Lour.	Khoi	Native	B	FT	FR	0.34	0.38	3.32	TJY0108
111.	Moringaceae	*Moringa oleifera* Lam.	Ma Rum	Introduced	C	FT, IF, LV	FP, VG	0.56	0.50	680.40	TJY0127
112.	Muntingiaceae	*Muntingia calabura* L.	Mak khop	Introduced	W	FT	FR	0.38	0.36	22.23	TJY0154
113.	Musaceae	*Musa acuminata* Colla	Kluai Hom	Native	W	FT	BV, FR, SW	0.72	0.70	324.00	TJY0145
114.	Musaceae	*Musa × paradisiaca* L.	Kluai Namwa	Introduced	C	FT	BV, FR, SW	0.74	0.70	333.00	TJY0146
115.	Myrtaceae	*Psidium guajava* L.	Farang	Introduced	C	FT	BV, FR	0.70	0.60	189.00	TJY0149

116.	Myrtaceae	*Syzygium antisepticum* (Blume) Merr. & L.M.Perry	Phak Mek	Native	C	LV	VG	0.64	0.66	103.68	TJY0150
117.	Myrtaceae	*Syzygium cumini* (L.) Skeels	Wa	Native	C	FT	FR	0.56	0.58	56.70	TJY0151
118.	Nelumbonaceae	*Nelumbo nucifera* Gaertn.	Bua Luang	Native	C	RT, RZ	SW, VG	0.48	0.52	97.20	TJY0128
119.	Nymphaeaceae	*Nymphaea nouchali* Burm.f.	Bua Phuean	Native	W	SM	VG	0.50	0.54	67.50	TJY0152
120.	Nymphaeaceae	*Nymphaea rubra* Roxb. ex Andrews	Bua Sai	Native	W	SM	VG	0.54	0.58	155.52	TJY0153
121.	Opiliaceae	*Melientha suavis* Pierre	Phak Wan	Native	B	FT, LV	VG	0.62	0.66	446.40	TJY0197
122.	Oxalidaceae	*Averrhoa carambola* L.	Mak fueang	Introduced	C	FT	FR	0.54	0.58	21.06	TJY0201
123.	Pandanaceae	*Pandanus amaryllifolius* Roxb. ex Lindl.	Toei Hom	Introduced	C	LV	BV, CF	0.52	0.50	121.68	TJY0148
124.	Passifloraceae	*Passiflora edulis* Sims	Kra Tokrok	Introduced	C	FT	BV, FR	0.36	0.40	14.04	TJY0129
125.	Phyllanthaceae	*Antidesma puncticulatum* Miq.	Ma Mao	Native	C	FT	BV, FR	0.34	0.38	19.89	TJY0098
126.	Phyllanthaceae	*Baccaurea ramiflora* Lour.	Ma Fai	Native	W	FT	FR	0.48	0.54	48.60	TJY0099
127.	Phyllanthaceae	*Bauhinia saccocalyx* Pierre	Siao	Native	B	LV	CF	0.32	0.34	24.96	TJY0100
128.	Phyllanthaceae	*Phyllanthus androgynus* (L.) Chakrab. & N.P.Balakr.	Phak Wan Ban	Native	C	LV	VG	0.30	0.32	30.38	TJY0101
129.	Phyllanthaceae	*Phyllanthus emblica* L.	Makham Pom	Native	C	FT	FR, MP	0.24	0.26	20.25	TJY0102
130.	Piperaceae	*Piper nigrum* L.	Phrik Thai	Introduced	W	FT	CF	0.50	0.54	135.00	TJY0142
131.	Piperaceae	*Piper sarmentosum* Roxb.	Chaphlu	Native	W	LV	CF, VG	0.54	0.56	349.92	TJY0143
132.	Plantaginaceae	*Limnophila aromatica* (Lam.) Merr.	Phak Kha-yaeng	Native	B	LV	CF, VG	0.44	0.48	213.84	TJY0124
133.	Poaceae	× *Thyrsocalamus liang* Sungkaew & W.L.Goh	Phai Liang	Native	B	SM	FP, VG	0.58	0.56	185.60	TJY0111
134.	Poaceae	*Bambusa bambos* (L.) Voss	Phai Pa	Native	B	SM	FP, VG	0.52	0.54	124.80	TJY0112
135.	Poaceae	*Bambusa spinosa* Roxb.	Phai Sisuk	Native	B	SM	FP, VG	0.54	0.52	129.60	TJY0113
136.	Poaceae	*Cymbopogon citratus* (DC.) Stapf	Takhrai	Introduced	C	LV	BV, CF, MP	0.74	0.76	799.20	TJY0114

137.	Poaceae	*Oryza sativa* L.	Khao	Introduced	C	SE	SF	1.00	1.00	400.00	TJY0115
138.	Poaceae	*Saccharum officinarum* L.	Oi	Introduced	C	SM	BV	0.92	0.90	132.48	TJY0116
139.	Poaceae	*Vietnamosasa ciliata* (A.Camus) T.Q.Nguyen	No Chot	Native	B	SM	FP, VG	0.48	0.54	115.20	TJY0117
140.	Poaceae	*Zea mays* L.	Khaophot	Introduced	C	SE	SF, SW	0.96	0.94	276.48	TJY0118
141.	Polygonaceae	*Persicaria odorata* (Lour.) Soják	Phak Phaeo	Native	C	LV	VG	0.54	0.56	145.80	TJY0123
142.	Rhamnaceae	*Ziziphus mauritiana* Lam.	Phutsa	Native	W	FT	FR	0.42	0.44	18.43	TJY0085
143.	Rhamnaceae	*Ziziphus oenopolia* (L.) Mill.	Lep Yiao	Native	W	FT	FR	0.32	0.34	9.36	TJY0086
144.	Rubiaceae	*Ixora chinensis* Lam.	Khem	Native	C	IF	VG	0.34	0.36	2.49	TJY0109
145.	Rubiaceae	*Morinda citrifolia* L.	Yo	Native	C	LV	VG	0.26	0.28	3.22	TJY0110
146.	Rutaceae	*Citrus × aurantiifolia* (Christm.) Swingle	Manao	Introduced	C	FT	BV, CF	0.94	0.92	846.00	TJY0119
147.	Rutaceae	*Citrus × aurantium* L.	Som Sa	Introduced	C	FT	BV, FR	0.60	0.66	64.80	TJY0120
148.	Rutaceae	*Citrus hystrix* DC.	Ma Krut	Native	C	LV	CF	0.70	0.68	226.80	TJY0121
149.	Rutaceae	*Citrus maxima* (Burm.) Merr.	Som O	Native	C	FT	FR	0.54	0.56	58.32	TJY0122
150.	Sapindaceae	*Dimocarpus longan* Lour.	Lamyai	Native	C	FT	BV, FR	0.52	0.54	56.16	TJY0087
151.	Sapindaceae	*Nephelium lappaceum* L.	Ngo	Native	C	FT	BV, FR	0.56	0.58	60.48	TJY0088
152.	Sapotaceae	*Chrysophyllum cainito* L.	Luk Nam-nom	Introduced	C	FT	FR	0.42	0.44	5.67	TJY0089
153.	Sapotaceae	*Manilkara zapota* (L.) P.Royen	La Mut	Introduced	C	FT	FR	0.54	0.54	21.87	TJY0090
154.	Solanaceae	*Capsicum annuum* L.	Phrik	Introduced	W	FT	CF	0.92	0.90	552.00	TJY0079
155.	Solanaceae	*Capsicum frutescens* L.	Phrik Khinu	Introduced	C	FT	CF	0.82	0.80	492.00	TJY0080
156.	Solanaceae	*Solanum lycopersicum* L.	Ma-khueathet	Introduced	C	FT	BV, VG	0.70	0.72	151.20	TJY0081
157.	Solanaceae	*Solanum melongena* L.	Makhuea Muang	Native	W	FT	VG	0.64	0.66	230.40	TJY0082
158.	Solanaceae	*Solanum torvum* Sw.	Makhuea Phuang	Introduced	C	FT	VG	0.56	0.58	201.60	TJY0083

159.	Solanaceae	*Solanum virginianum* L.	Makhuea Pro	Introduced	C	FT	VG	0.84	0.88	453.60	TJY0084
160.	Vitaceae	*Ampelocissus martini* Planch.	I Goy	Native	W	FT	FR	0.22	0.26	3.71	TJY0050
161.	Zingiberaceae	*Alpinia galanga* (L.) Willd.	Kha	Native	C	RZ	CF	0.40	0.44	259.20	TJY0040
162.	Zingiberaceae	*Alpinia siamensis* K.Schum.	Kha	Native	C	RZ	CF	0.78	0.76	505.44	TJY0041
163.	Zingiberaceae	*Boesenbergia rotunda* (L.) Mansf.	Kra Chai	Native	C	RZ	CF	0.68	0.66	440.64	TJY0042
164.	Zingiberaceae	*Curcuma angustifolia* Roxb.	Kra Chiao Daeng	Native	B	IF	VG	0.62	0.60	62.78	TJY0043
165.	Zingiberaceae	*Curcuma comosa* Roxb.	Wan Chak Motluk	Native	C	RZ	MP	0.26	0.28	21.45	TJY0044
166.	Zingiberaceae	*Curcuma longa* L.	Khamin	Introduced	C	RZ	CF, MP	0.64	0.66	108.00	TJY0045
167.	Zingiberaceae	*Curcuma singularis* Gagnep.	Kra Chiao	Native	B	IF	VG	0.50	0.52	16.88	TJY0046
168.	Zingiberaceae	*Kaempferia marginata* Carey ex Roscoe	Pro Hom	Native	C	LV	VG	0.32	0.34	12.96	TJY0047
169.	Zingiberaceae	*Zingiber montanum* (J.Koenig) Link ex A.Dietr.	Wan Fai	Native	C	LV, RZ	MP, VG	0.22	0.24	74.25	TJY0048
170.	Zingiberaceae	*Zingiber officinale* Roscoe	Khing	Introduced	C	FT, RT, RZ	BV, CF, MP, VG	0.38	0.40	307.80	TJY0049

Abbreviation: Resource: cultivated and wild (B), cultivated (C), wild (W). Used part: root or storage root (RT), bark (BK), bulb (BU), corm (CM), fruit (FT), inflorescence (IF), leaves (LV), rhizome (RZ), seed (SE), stem or shoot (SM), tuber (TB), vine (VN), whole plant (WP). Utilization: beverages (BV), condiments and flavoring (CF), fermented or preserved (FP), fruit (FR), medicinal edible plant (MP), staple food (SF), sweets, desserts, or snacks (SW), vegetable (VG).

Table S2. Fidelity Level (FL) evaluation of edible plants in Mueang District, Yasothon Province.

Scientific name	N_p	N_t	FL	Used parts	Preparation	Therapeutic uses and health benefits	Therapeutic categories
Achyranthes aspera L.	6	11	54.55	Whole plant	Decoction	Traditionally used to reduce fever	Infection, Parasite and Immune system
	5	11	45.45	Root	Decoction	Traditionally used to relieve bloating, flatulence, and chest discomfort associated with indigestion	Gastrointestinal
Andrographis paniculata (Burm.f.) Wall. ex Nees	11	16	68.75	Leaves	Decoction	Traditionally used to alleviate common febrile conditions, including symptoms such as headache and fever, commonly associated with colds and influenza.	Infection, Parasite and Immune system
	5	16	31.25	Whole plant	Decoction	Traditionally used to treat gastrointestinal infections by suppressing the growth of microorganisms that cause abdominal pain, diarrhea, dysentery, and related digestive disorders.	Gastrointestinal
Barleria prionitis L.	8	12	66.67	Whole plant	Decoction	Traditionally used as a diuretic to promote urination	Obstetrics, Gynaecology and urinary disorders
	4	12	33.33	Whole plant	Decoction	Traditionally used to alleviate weakness in the limbs and reduce symptoms of arthritis or joint inflammation.	Musculoskeletal and joint diseases
Blumea balsamifera (L.) DC.	5	9	55.56	Leaves	Decoction	Traditionally used to reduce fever	Infection, Parasite and Immune system
	2	9	22.22	Leaves	Decoction	Traditionally used to expel intestinal parasites	Infection, Parasite and Immune system
	2	9	22.22	Leaves	Decoction	Traditionally used to relieve flatulence, abdominal bloating, and stomach discomfort	Gastrointestinal
Centella asiatica (L.) Urb.	29	29	100.00	Leaves	Squeeze	Traditionally used to relieve internal bruising and inflammation	Musculoskeletal and joint diseases
Cryptolepis buchananii R.Br. ex Roem. & Schult.	10	16	62.50	Vine	The vine is dried, pound into powder, and mixed	Traditionally used to relieve bruising and subcutaneous bleeding	Musculoskeletal and joint diseases

					with alcohol before use		
	6	16	37.50	Leaves	Decoction	Traditionally used to relieve muscle fatigue, relax tendons, nourish and strengthen the tendinous system, and treat tendon disorders	Musculoskeletal and joint diseases
Curcuma comosa Roxb.	9	13	69.23	Rhizome	Infused in hot wate	Traditionally employed to assist uterine involution after childbirth, correct uterine dysfunction, and alleviate uterine cramps or pain	Obstetrics, Gynaecology and urinary disorders
	4	13	30.77	Rhizome	Infused in hot wate	Traditionally employed to regulate irregular menstruation, alleviate dysmenorrhea, manage abnormal vaginal discharge (leucorrhea), and support the expulsion of lochia after childbirth	Obstetrics, Gynaecology and urinary disorders
Curcuma longa L.	18	32	56.25	Rhizome	Dried under sunlight, ground into fine powder, mixed with honey, and formed into pills	Traditionally used to relieve bloating, flatulence, abdominal discomfort, and indigestion	Gastrointestinal
	14	32	43.75	Rhizome	Dried under sunlight, ground into fine powder, mixed with honey, and formed into pills	Traditionally used to treat dyspepsia (indigestion), including symptoms such as bloating, nausea, and abdominal discomfort	Gastrointestinal
Cymbopogon citratus (DC.) Stapf	22	37	59.46	Leaves	Decoction	Traditionally used to reduce fever	Infection, Parasite and Immune system
	15	37	40.54	Leaves	Decoction	Traditionally employed to alleviate abdominal discomfort, including bloating and flatulence, and to support healthy digestion	Gastrointestinal
Ellipanthus tomentosus Kurz	6	10	60.00	Stem	Decoction	Traditionally employed to alleviate abdominal discomfort and reduce spasmodic contractions of the abdominal muscles	Gastrointestinal

	4	10	40.00	Stem	Decoction	Traditionally employed to alleviate urinary tract conditions, act as a natural diuretic, and support the treatment of kidney dysfunction	Obstetrics, Gynaecology and urinary disorders
Euphorbia hirta L.	10	14	71.43	Stem	Decoction	Traditionally employed to alleviate dysuria (painful or difficult urination) and reduce blood in the urine (hematuria)	Obstetrics, Gynaecology and urinary disorders
	4	14	28.57	Stem	Decoction	Traditionally employed to support the treatment of gonorrhea	Infection, Parasite and Immune system
Gloriosa simplex L.	8	14	57.14	Root	Decoction	Traditionally employed to reduce excessive phlegm, alleviate mucus buildup, and support respiratory function	Reproductive system
	6	14	42.86	Root	Decoction	Traditionally employed to alleviate knee joint pain, reduce inflammation, and relieve swelling associated with musculoskeletal disorders	Musculoskeletal and joint diseases
Hellenia speciosa (J.Koenig) S.R.Dutta	15	26	57.69	Root	Decoction	Traditionally employed to support digestive function and improve gastrointestinal efficiency	Gastrointestinal
	6	26	23.08	Root	Decoction	Traditionally employed as a remedy for various skin disorders, including rashes, itching, and inflammation	Skin system
	5	26	19.23	Stem	Decoction	Traditionally employed as a natural diuretic to promote urination and to alleviate symptoms of bladder inflammation (cystitis)	Obstetrics, Gynaecology and urinary disorders
Mansonia gagei J.R.Drumm.	8	15	53.33	Stem	Decoction	Traditionally employed to promote the expulsion of gas from the intestines, ease gastrointestinal bloating and discomfort, and relieve colicky or cramping abdominal pain	Gastrointestinal
	7	15	46.67	Stem	Decoction	Traditionally used to reduce fever	Infection, Parasite and Immune system
Orthosiphon aristatus (Blume) Miq.	10	14	71.43	Leaves	Decoction	Traditionally used to lower blood pressure and manage symptoms of diabetes	Nutrition and blood
	4	14	28.57	Fruit	Decoction	Traditionally employed to promote the healing of gastric and intestinal ulcers by protecting and restoring the mucosal lining	Gastrointestinal

Phyllanthus emblica L.	7	12	58.33	Fruit	Consumed	Traditionally employed to alleviate coughing, reduce throat irritation, and promote the expectoration of mucus	Gastrointestinal
	5	12	41.67	Fruit	Consumed	Traditionally employed as a natural remedy to alleviate constipation and promote regular bowel movements.	Gastrointestinal
Pluchea indica (L.) Less.	9	16	56.25	Whole plant	Decoction	Traditionally used to relieve painful or difficult urination	Obstetrics, Gynaecology and urinary disorders
	7	16	43.75	Leaves	Decoction	Traditionally used to lower blood pressure and manage symptoms of diabetes	Nutrition and blood
Salacia chinensis L.	8	16	50.00	Stem	Decoction	Traditionally employed to relieve constipation, promote bowel movements, and alleviate symptoms associated with upward-moving wind, such as nausea or dizziness	Gastrointestinal
	8	16	50.00	Stem	Decoction	Traditionally employed to alleviate muscular aches and pains, improve circulation, and relax muscles and tendons	Musculoskeletal and joint diseases
Streptocaulon juventas (Lour.) Merr.	10	11	90.91	Whole plant	Decoction	Traditionally used to promote postpartum recovery by expelling lochia and impure blood in women	Obstetrics, Gynaecology and urinary disorders
	1	11	9.09	Root	Decoction	Traditionally used to reduce fever	Infection, Parasite and Immune system
Suregada multiflora (A.Juss.) Baill.	8	12	66.67	Bark	Decoction	Traditionally employed to relieve various skin disorders, particularly fungal infections such as ringworm (tinea corporis) and other forms of dermatomycosis	Skin system
	4	12	33.33	Bark	Decoction	Traditionally employed as a natural purgative and mild laxative to promote bowel movements, and also used to nourish and strengthen the gums, reduce gingival inflammation, and support overall oral health and dental durability	Gastrointestinal
Thunbergia laurifolia Lindl.	9	15	60.00	Leaves	Squeeze	Traditionally used to reduce fever and neutralize poisons from plants, mushrooms, venomous animals, and intoxicating substances.	Poisoning and Toxicology

	6	15	40.00	Leaves	Pounded into a fine paste and mixed with rice-washing water	Traditionally used to help regulate blood sugar levels in diabetic patients	Nutrition and blood
Zingiber montanum (J.Koenig) Link ex A.Dietr.	7	11	63.64	Rhizome	Decoction	Traditionally used to eliminate impure blood and regulate menstruation in women	Obstetrics, Gynaecology and urinary disorders
	4	11	36.36	Rhizome	Decoction	Traditionally used to relieve bloating, abdominal pain, constipation, vomiting, and toothache	Gastrointestinal
Zingiber officinale Roscoe	10	19	52.63	Rhizome	Consumed	Traditionally used as a general tonic to support digestive fire and restore elemental balance in the body	Gastrointestinal
	6	19	31.58	Fruit	Decoction	Traditionally used to relieve dry throat and sore throat	Infection, Parasite and Immune system
	3	19	15.79	Root	Decoction	Traditionally used to reduce phlegm and clear respiratory passages	Infection, Parasite and Immune system

Table S3. Cultural Food Significance Index (CFSI) evaluation of Edible Plants in Mueang District, Yasothon Province.

Scientific name	QI	AI	FUI	PUI	MFFI	TSAI	FMRI	CFSI
Hellenia speciosa (J.Koenig) S.R.Dutta	52	34	2	3.25	1	7.5	4	3447.60
Allium cepa L.	90	4	5	4.5	1	9	3	2187.00
Citrus × aurantiifolia (Christm.) Swingle	94	4	5	1.5	1	10	3	846.00
Cymbopogon citratus (DC.) Stapf	74	4	5	1.5	1	9	4	799.20
Senna siamea (Lam.) H.S.Irwin & Barneby	88	4	5	1.5	1	10	3	792.00
Ipomoea aquatica Forssk.	86	4	5	2.5	1	9	2	774.00
Allium sativum L.	92	4	5	1.5	1	9	3	745.20
Moringa oleifera Lam.	56	3	4	3.75	1	9	3	680.40
Tamarindus indica L.	64	4	5	3.75	0.5	9	3	648.00
Ocimum tenuiflorum L.	78	4	5	1.5	1	9	3	631.80
Tiliacora triandra (Colebr.) Diels	68	4	5	1.5	1	10	3	612.00
Azadirachta indica A.Juss.	56	4	4	2.25	1	10	3	604.80

Capsicum annuum L.	92	4	5	1.5	1	10	2	552.00
Lathyrus oleraceus Lam.	72	4	4	3	1	7.5	2	518.40
Alpinia siamensis K.Schum.	78	4	4	1.5	1	9	3	505.44
Daucus carota L.	76	4	4	1.5	1.5	9	2	492.48
Capsicum frutescens L.	82	4	5	1.5	1	10	2	492.00
Luffa aegyptiaca Mill.	84	4	5	1.5	1	9	2	453.60
Solanum virginianum L.	84	4	4	1.5	1	7.5	3	453.60
Melientha suavis Pierre	62	4	3	3	1	10	2	446.40
Cucurbita maxima Duchesne	82	4	5	1.5	1	9	2	442.80
Boesenbergia rotunda (L.) Mansf.	68	4	4	1.5	1	9	3	440.64
Sesbania grandiflora (L.) Poir.	54	4	5	2.25	1	9	2	437.40
Coriandrum sativum L.	90	4	4	3	0.5	10	2	432.00
Oryza sativa L.	100	4	5	1	1	10	2	400.00
Oroxylum indicum (L.) Kurz	58	4	3	3.75	1	7.5	2	391.50
Luffa acutangula (L.) Roxb.	90	4	4	1.5	1	9	2	388.80
Cratoxylum formosum (Jack) Benth. & Hook.f. ex Dyer	78	4	4	1.5	1	10	2	374.40
Brassica oleracea L. cv. "Kalam Pli"	86	4	4	1.5	1	9	2	371.52
Piper sarmentosum Roxb.	54	4	4	1.5	1	9	3	349.92
Brassica oleracea L. cv. "Phak Khana"	80	4	4	1.5	1	9	2	345.60
Trichosanthes cucumerina L.	78	4	4	1.5	1	9	2	336.96
Musa × paradisiaca L.	74	4	5	1.5	0.5	10	3	333.00
Allium tuberosum Rottler ex Spreng.	76	4	4	1.5	1	9	2	328.32
Brassica rapa L.	76	4	4	1.5	1	9	2	328.32
Musa acuminata Colla	72	4	5	1.5	0.5	10	3	324.00
Zingiber officinale Roscoe	38	3	3	3	1	7.5	4	307.80
Ocimum × africanum Lour.	70	4	5	1.5	0.5	9	3	283.50
Cocos nucifera L.	78	4	4	2.5	0.5	9	2	280.80
Zea mays L.	96	4	4	1	1	9	2	276.48

Momordica charantia L.	76	4	4	3	0.5	7.5	2	273.60
Centella asiatica (L.) Urb.	58	3	4	1.5	1	6.5	4	271.44
Carica papaya L.	90	4	5	1.5	0.5	10	2	270.00
Lagenaria siceraria (Molina) Standl.	80	4	3	1.5	1	9	2	259.20
Alpinia galanga (L.) Willd.	40	4	4	1.5	1	9	3	259.20
Eryngium foetidum L.	70	4	4	1.5	1	7.5	2	252.00
Raphanus raphanistrum subsp. *sativus* (L.) Domin	58	4	4	1.5	1	9	2	250.56
Ipomoea batatas (L.) Lam.	74	4	3	1.5	1	9	2	239.76
Coccinia grandis (L.) Voigt	74	4	3	1.5	1	9	2	239.76
Solanum melongena L.	64	4	4	1.5	1	7.5	2	230.40
Melissa officinalis L.	56	4	5	1.5	0.5	9	3	226.80
Citrus hystrix DC.	70	4	3	1.5	1	9	2	226.80
Limnophila aromatica (Lam.) Merr.	44	4	3	1.5	1	9	3	213.84
Spondias pinnata (L.f.) Kurz	78	4	3	1.5	1	7.5	2	210.60
Arachis hypogaea L.	72	4	4	1	1	9	2	207.36
Solanum torvum Sw.	56	4	4	1.5	1	7.5	2	201.60
Brassica juncea (L.) Czern.	60	4	3	1.5	1	9	2	194.40
Neptunia oleracea Lour.	70	4	3	1.5	1	7.5	2	189.00
Psidium guajava L.	70	4	5	1.5	0.5	9	2	189.00
× *Thyrsocalamus liang* Sungkaew & W.L.Goh	58	4	4	1	1	10	2	185.60
Apium sellowianum H.Wolff	84	4	4	1.5	0.5	9	2	181.44
Cucumis sativus L.	84	4	4	1.5	0.5	9	2	181.44
Sicyos edulis Jacq.	56	4	3	1.5	1	9	2	181.44
Benincasa hispida (Thunb.) Cogn.	66	4	3	1.5	1	7.5	2	178.20
Anethum graveolens L.	80	4	4	1.5	0.5	9	2	172.80
Leucaena leucocephala (Lam.) de Wit	64	4	3	3	0.5	7.5	2	172.80
Psophocarpus tetragonolobus (L.) DC.	78	4	4	1.5	0.5	9	2	168.48
Lactuca sativa L.	82	3	3	1.5	1	7.5	2	166.05

Mangifera indica L.	90	4	3	1.5	0.5	10	2	162.00
Artocarpus heterophyllus Lam.	72	4	4	1.5	0.5	9	2	155.52
Nymphaea rubra Roxb. ex Andrews	54	4	4	1	1	9	2	155.52
Senegalia pennata (L.) Maslin	56	4	5	1.5	0.5	9	2	151.20
Solanum lycopersicum L.	70	4	4	1.5	0.5	9	2	151.20
Persicaria odorata (Lour.) Soják	54	3	3	1.5	1	10	2	145.80
Amaranthus blitum L.	70	3	3	1.5	1	7.5	2	141.75
Annona squamosa L.	84	4	3	1.5	0.5	9	2	136.08
Piper nigrum L.	50	4	2	1.5	1	7.5	3	135.00
Amaranthus viridis L.	66	3	3	1.5	1	7.5	2	133.65
Saccharum officinarum L.	92	4	4	1	0.5	9	2	132.48
Limnocharis flava (L.) Buchenau	80	4	3	0.75	1	9	2	129.60
Bambusa spinosa Roxb.	54	4	3	1	1	10	2	129.60
Pluchea indica (L.) Less.	32	2	2	4.5	1	5.5	4	126.72
Citrullus lanatus (Thunb.) Matsum. & Nakai	78	4	3	1.5	0.5	9	2	126.36
Wolffia globosa (Roxb.) Hartog & Plas	70	3	2	1.5	1	10	2	126.00
Bambusa bambos (L.) Voss	52	4	3	1	1	10	2	124.80
Pandanus amaryllifolius Roxb. ex Lindl.	52	4	2	1.5	1	6.5	3	121.68
Cucumis melo L.	72	4	3	1.5	0.5	9	2	116.64
Vietnamosasa ciliata (A.Camus) T.Q.Nguyen	48	4	3	1	1	10	2	115.20
× *Brassarda juncea* (L.) Su Liu & Z.H.Feng	62	4	2	1.5	1	7.5	2	111.60
Cleome gynandra L.	68	3	2	3	0.5	9	2	110.16
Curcuma longa L.	64	3	1	1.5	1	7.5	5	108.00
Garcinia mangostana L.	64	4	3	1.5	0.5	9	2	103.68
Syzygium antisepticum (Blume) Merr. & L.M.Perry	64	3	4	1.5	0.5	9	2	103.68
Vigna radiata (L.) R.Wilczek	48	4	4	1	1	6.5	2	99.84
Ananas comosus (L.) Merr.	60	4	3	1.5	0.5	9	2	97.20
Nelumbo nucifera Gaertn.	48	3	3	1.5	1	7.5	2	97.20

Cullenia ceylanica (Gardner) Wight ex K.Schum.	70	3	3	1.5	0.5	10	2	94.50
Pachyrhizus erosus (L.) Urb.	58	4	3	1.5	0.5	9	2	93.96
Orthosiphon aristatus (Blume) Miq.	28	2	2	3	1	5.5	5	92.40
Vigna mungo (L.) Hepper	58	4	3	1	1	6.5	2	90.48
Vigna unguiculata subsp. *sesquipedalis* (L.) Verdc.	56	4	4	1	0.5	9	2	80.64
Andrographis paniculata (Burm.f.) Wall. ex Nees	32	2	1	4.5	1	5.5	5	79.20
Zingiber montanum (J.Koenig) Link ex A.Dietr.	22	3	1	3	1	7.5	5	74.25
Dolichandrone serrulata (Wall. ex DC.) Seem.	54	4	3	0.75	1	7.5	2	72.90
Lansium domesticum Corrêa	58	3	3	1.5	0.5	9	2	70.47
Nymphaea nouchali Burm.f.	50	3	3	1	1	7.5	2	67.50
Elaeocarpus hygrophilus Kurz	60	4	2	1.5	0.5	9	2	64.80
Citrus × *aurantium* L.	60	4	2	1.5	0.5	9	2	64.80
Curcuma angustifolia Roxb.	62	3	3	0.75	0.5	10	3	62.78
Careya arborea Roxb.	50	3	3	1.5	0.5	9	2	60.75
Epicharis parasitica (Osbeck) Mabb.	50	3	3	1.5	0.5	9	2	60.75
Nephelium lappaceum L.	56	4	2	1.5	0.5	9	2	60.48
Sesbania javanica Miq.	38	4	4	0.75	1	6.5	2	59.28
Thunbergia laurifolia Lindl.	30	2	2	1.5	1	6.5	5	58.50
Citrus maxima (Burm.) Merr.	54	4	2	1.5	0.5	9	2	58.32
Basella alba L.	56	3	3	0.75	1	7.5	2	56.70
Punica granatum L.	56	3	3	1.5	0.5	7.5	2	56.70
Syzygium cumini (L.) Skeels	56	3	3	1.5	0.5	7.5	2	56.70
Dimocarpus longan Lour.	52	4	2	1.5	0.5	9	2	56.16
Telosma cordata (Burm.f.) Merr.	54	3	3	0.75	1	7.5	2	54.68
Achyranthes aspera L.	22	2	1	4.5	1	5.5	5	54.45
Amorphophallus brevispathus Gagnep.	50	3	2	1	1	9	2	54.00
Baccaurea ramiflora Lour.	48	3	3	1.5	0.5	7.5	2	48.60
Clitoria ternatea L.	68	4	2	0.75	0.5	7.5	3	45.90

Salacca wallichiana Mart.	50	3	2	1.5	0.5	9	2	40.50
Barleria prionitis L.	24	2	1	3	1	5.5	5	39.60
Cassia fistula L.	44	3	2	0.75	1	6.5	3	38.61
Abelmoschus esculentus (L.) Moench	44	3	3	1.5	0.5	6.5	2	38.61
Butea monosperma (Lam.) Kuntze	56	2	3	0.75	1	7.5	2	37.80
Salacia chinensis L.	32	2	2	1	1	5.5	5	35.20
Colocasia esculenta (L.) Schott	44	3	2	1	1	6.5	2	34.32
Calamus viminalis Willd.	42	2	2	1	1	10	2	33.60
Oenanthe javanica (Blume) DC.	56	3	2	1.5	0.5	6.5	2	32.76
Pithecellobium dulce (Roxb.) Benth.	54	3	2	1.5	0.5	6.5	2	31.59
Bombax anceps Pierre	52	3	2	0.75	1	6.5	2	30.42
Marsilea crenata C.Presl	52	2	3	1.5	0.5	6.5	2	30.42
Phyllanthus androgynus (L.) Chakrab. & N.P.Balakr.	30	3	1	1.5	1	7.5	3	30.38
Irvingia malayana Oliv. ex A.W.Benn.	48	2	2	1.5	0.5	10	2	28.80
Streptocaulon juventas (Lour.) Merr.	22	1	1	4.5	1	5.5	5	27.23
Morus alba L.	38	2	3	1.5	0.5	7.5	2	25.65
Bauhinia saccocalyx Pierre	32	2	2	1.5	1	6.5	2	24.96
Muntingia calabura L.	38	3	2	1.5	0.5	6.5	2	22.23
Cryptolepis buchananii R.Br. ex Roem. & Schult.	32	1	1	2.5	1	5.5	5	22.00
Manilkara zapota (L.) P.Royen	54	3	1	1.5	0.5	9	2	21.87
Carissa carandas L.	56	2	2	1.5	0.5	6.5	2	21.84
Myriopteron extensum (Wight) K.Schum.	28	2	2	1	1	6.5	3	21.84
Curcuma comosa Roxb.	26	2	1	1.5	1	5.5	5	21.45
Averrhoa carambola L.	54	2	2	1.5	0.5	6.5	2	21.06
Phyllanthus emblica L.	24	3	1	1.5	0.5	7.5	5	20.25
Antidesma puncticulatum Miq.	34	3	2	1.5	0.5	6.5	2	19.89
Ziziphus mauritiana Lam.	42	3	1	1.5	0.5	6.5	3	18.43
Parkia speciosa Hassk.	44	3	2	1	0.5	6.5	2	17.16

Curcuma singularis Gagnep.	50	3	1	0.75	0.5	10	3	16.88
Passiflora edulis Sims	36	2	2	1.5	0.5	6.5	2	14.04
Kaempferia marginata Carey ex Roscoe	32	2	1	1.5	0.5	9	3	12.96
Gloriosa simplex L.	28	1	1	1.5	1	5.5	5	11.55
Ziziphus oenopolia (L.) Mill.	32	2	1	1.5	0.5	6.5	3	9.36
Mansonia gagei J.R.Drumm.	30	1	1	1	1	5.5	5	8.25
Ficus racemosa L.	40	1	2	1.5	0.5	6.5	2	7.80
Euphorbia hirta L.	28	1	1	1	1	5.5	5	7.70
Blumea balsamifera (L.) DC.	18	1	1	1.5	1	5.5	5	7.43
Polyalthia debilis (Pierre) Finet & Gagnep.	38	1	2	1.5	0.5	6.5	2	7.41
Suregada multiflora (A.Juss.) Baill.	24	1	1	1	1	5.5	5	6.60
Chrysophyllum cainito L.	42	1	1	1.5	0.5	9	2	5.67
Ellipanthus tomentosus Kurz	20	1	1	1	1	5.5	5	5.50
Polyalthia evecta (Pierre) Finet & Gagnep.	28	1	2	1.5	0.5	6.5	2	5.46
Ampelocissus martini Planch.	22	1	1	1.5	0.5	7.5	3	3.71
Streblus asper Lour.	34	1	1	1.5	0.5	6.5	2	3.32
Morinda citrifolia L.	26	1	1	1.5	0.5	5.5	3	3.22
Ixora chinensis Lam.	34	1	1	0.75	0.5	6.5	3	2.49

Table S4. Analysis and Calculation of the Cultural Food Significance Index (CFSI).

Classifications	Index	Index value
**AI (Availability Index)**	**Availability**	
	Very common	4.00
	Common	3.00
	Middle	2.00
	Rare	1.00
	**Localisation of use index value**	

	Ubiquitary	=
	Localised	-0.50
	Very localised	-0.10
**FUI (Frequency of Utilization Index)**	**Utilisation Frequency**	
	＞Once/week	5.00
	Once/week	4.00
	Once/month	3.00
	＞once/year but＜once/month	2.00
	Once/year	1.00
	No longer used during the past 30 years	0.50
**PUI (Parts Used Index)**	**Part used**	
	bark	1.00
	roots or rootstocks	1.50
	roots, only younger parts	1.00
	bulbs	1.50
	stems	1.00
	leaves	1.50
	leaves stalks	1.00
	young whorls of leaves	1.00
	leaves with a few stems	2.00
	shoots	1.25
	shoots, only younger parts	0.75
	buds	0.75
	flowers	0.75
	receptacles	0.75
	fruits	1.50
	seeds	1.00
	whole aerial parts	3.00

	whole aerial parts of very young plants	2.00
	caps (mushrooms)	1.50
	whole fruiting body (mushrooms)	2.00
**MFFI** (**Multi**-F**unctional Food Use Index**	**Usage**	
	Raw, as snack	0.50
	Raw, in salads	1.50
	Fried in fat, without or with beaten eggs ("Frittata")	1.00
	Boiled	1.00
	Boiled, then stewed of fried	1.50
	Boiled, than as stuffing for diverse preparations (pies, "tortelli"...)	1.50
	Soups (mixtures)	0.75
	Stewed	1.00
	Roasted	1.00
	Condiment	1.00
	Condiment for restricted purposes	0.75
	Jams or Jellies	1.00
	Syrups	1.00
	(Usage in mixtures)	(-0.5)
**TASI** (**Taste Score Appreciation Index**	**Taste Appreciation**	
	Best	10.00
	Very good	9.00
	Good	7.50
	Fair	6.50
	Poor	5.50
	Terrible	4.00
**FMRI** (**Food-Medicinal Role Index**)	**Role as Food-Medicine**	
	Very high (“that food is a medicine!”)	5.00

	High ("that food is quite a medicine", with clear specification of the treated affections)	4.00
	Middle-high ("that food is very healthy")	3.00
	Middle-low ("that food is healthy", no specification of a particular therapeutic action)	2.00
	Not recognized	1.00
